# Arterial occlusion duration affects the cuff-induced hyperemic response in skeletal muscle BOLD perfusion imaging as shown in young healthy subjects

**DOI:** 10.1007/s10334-023-01105-y

**Published:** 2023-06-17

**Authors:** Jonathan Arvidsson, Stefanie Eriksson, Edvin Johansson, Kerstin Lagerstrand

**Affiliations:** 1 Department of Medical Radiation Sciences, Institute of Clinical Sciences, Sahlgrenska Academy, University of Gothenburg, Gothenburg, Sweden; 2 Department of Medical Physics and Biomedical Engineering, Sahlgrenska University Hospital, Gothenburg, Sweden; 3 Antaros Medical, Mölndal, Sweden

**Keywords:** BOLD MRI, Semi-quantitative, ${\mathrm{T}}_{2}^{*}$, Ischemia, Hyperemia

## Abstract

**Objective:**

Dynamic BOLD MRI with cuff compression, inducing ischemia and post-occlusive hyperemia in skeletal muscle, has been pointed out as a potential diagnostic tool to assess peripheral limb perfusion. The objective was to explore the robustness of this technique and its sensitivity to the occlusion duration.

**Materials and methods:**

BOLD images were acquired at 3 T in 14 healthy volunteers. ${\mathrm{T}}_{2}^{*}$-imaging with 5- and 1.5-min occlusions were acquired and several semi-quantitative BOLD parameters were derived from ROI-based ${\mathrm{T}}_{2}^{*}$-time curves. Differences in parameters from the two different occlusion durations were evaluated in the gastrocnemius and soleus muscles using non-parametrical tests. Intra- and inter-scan repeatability were evaluated with coefficient of variation.

**Results:**

Longer occlusion duration resulted in an increased hyperemic signal effect yielding significantly different values (*p* < 0.05) in gastrocnemius for all parameters describing the hyperemic response, and in soleus for two of these parameters. Specifically, 5-min occlusion yielded steeper hyperemic upslope in gastrocnemius (41.0%; *p* < 0.05) and soleus (59.7%; *p* = 0.03), shorter time to half peak in gastrocnemius (46.9%; *p* = 0.00008) and soleus (33.5%; *p* = 0.0003), and shorter time to peak in gastrocnemius (13.5%; *p* = 0.02). Coefficients of variation were lower than percentage differences that were found significant.

**Discussion:**

Findings show that the occlusion duration indeed influences the hyperemic response and thus should play a part in future methodological developments.

**Supplementary Information:**

The online version contains supplementary material available at 10.1007/s10334-023-01105-y.

## Introduction

Blood oxygenation level dependent (BOLD) magnetic resonance imaging (MRI) is sensitive to changes in the ratio between oxygenated and deoxygenated hemoglobin in the blood [1] and, as such, can reflect alterations in perfusion. The method has since its discovery become an invaluable tool for mapping activated neurons in the brain with many applications [2, 3]. In the last decade, skeletal muscle BOLD MRI has been identified as a tool for assessments of physiological and pathophysiological alterations in peripheral limb perfusion [4]. The technique has been shown to differentiate between healthy controls and patients with, for example, peripheral artery occlusive disease PAOD [5–9], critical limb ischemia (CLI) [10], type 2 diabetes [11], and systemic sclerosis [12]. The technique has also been shown to reflect positive results of revascularization in patients with PAOD and critical limb ischemia [10, 13] as well as medical treatment of patients with type 2 diabetes [14].

Studies indicate that, for example, age [15–17], sex [18], smoking habits [19], and fitness level [20] have an effect on the BOLD response in skeletal muscle. Most of these studies have been performed on calf muscles, where several approaches to provoke BOLD changes in the skeletal muscle have been demonstrated [4, 16]. One of these approaches is cuff compression-induced ischemia of the calf muscle with subsequent post-occlusion reactive hyperemia, which was first demonstrated by Toussaint et al. [21] and has been found to have the best reproducibility [16]. During occlusion, the deoxyhemoglobin in the calf muscle will increase during ischemia. The effect can be detected by BOLD MRI as a reduction in the tissue transverse relaxation time (${\mathrm{T}}_{2}^{*}$) from the baseline resting state value. After subsequent reactive hyperemia, ${\mathrm{T}}_{2}^{*}$ will rapidly increase, resulting in a peak value about 30–60 s after cuff deflation [4], after which ${\mathrm{T}}_{2}^{*}$ will return to a value close to baseline. During the last decades, the technique has been used in several studies [22], and while it continues to show promise as a technique for differentiation of levels of PAD and systemic sclerosis, there is currently no clear convergence toward reference values in healthy subjects. Probably this is due to variations in methodology. To enable implementation of skeletal muscle BOLD MRI in the clinical setting, guidelines on specific scan and post-processing protocols are needed to ensure high levels of accuracy and precision in the derived parameters.

One apparent methodologic difference between studies using the cuff compression-induced BOLD MRI technique is the duration of the arterial occlusion. The literature reports a large range of occlusion durations (3 [23], 4 [24], 5 [16, 20], 6 [5, 25], and 20 [26] minutes) with large differences in BOLD parameters in healthy subjects. Further, the literature points toward increased hyperemic effects, such as longer periods of increased blood flow upon cuff deflation as a result of longer occlusion durations [27–29]. Langham et al. reported a marginal increase in the venous oxygen saturation after 5-min occlusion compared to 3 min [30]. Bartlett et al. show differences in microvascular and macrovascular measures between 3- and 6-min occlusions, both of which should contribute to the BOLD response [29]. Based on these studies, we hypothesize that the large variance in assessed parameters between studies can partly be explained by differences in used occlusion duration.

## Materials and methods

In short, the robustness of the cuff compression-induced skeletal muscle BOLD MRI technique was evaluated by studying the reproducibility and sensitivity of the compression paradigm applied to two different calf muscles: gastrocnemius and soleus, in healthy subjects in regard to a number of aspects. The evaluations included repeated measurements within the same imaging session and between scanning sessions, executed at least 1 week apart. Furthermore, the sensitivity of derived parameters to the cuff compression paradigm were evaluated by comparing a long compression of 5 min to a short one of 1.5 min. Finally, inter- and intra-operator reproducibility were assessed based on the derived parameters with respect to two different operators ROI segmentations and curve fitting validations.

### Study participants

Fifteen healthy individuals volunteered to participate in this study. One subject was excluded because the circumference of the thigh of the examined leg was large, and therefore, a larger size tourniquet cuff was needed for this participant, leaving a total of 14 included subjects (age: 22–39 years, mean 29 years; 6 males).

The inclusion criteria for this study were < 40 years, healthy at the time of the examination, and with no previous history of cardiovascular disease. Also, the arm systolic blood pressure measured in a sitting position just before each scanning session needed to be < 140 mmHg. Participants were instructed to avoid strenuous exercise 24 h prior to every scanning session. All participants gave written informed consent before participation. This study was approved by the Västra Götaland research ethics committee (2019-03928/1157-17; 2018-02-13).

### Compression paradigm for reactive hyperemia

**Fig. 1:**
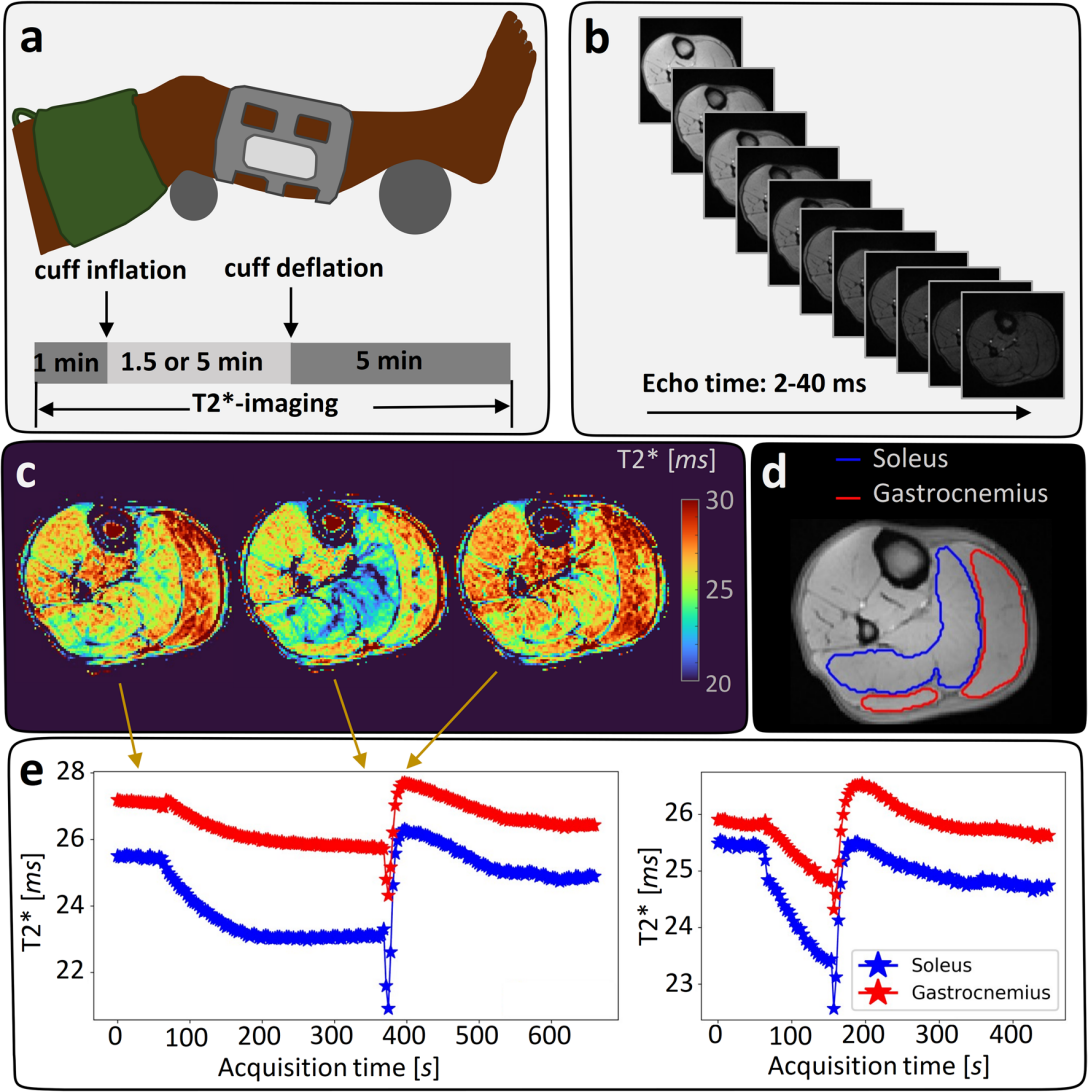
**a** Positioning of the examined leg with a blood pressure cuff around the thigh and a flex coil around the calf (above). Cuff compression paradigm during ${\mathrm{T}}_{2}^{*}$-imaging (below). After 1 min of baseline imaging the cuff was inflated followed by a 1.5- or 5-min ischemic period after which the cuff was deflated and followed by a 5-min hyperemic period. **b** ${\mathrm{T}}_{2}^{*}$-weighted multi-echo gradient-echo with equidistant echo times between 2 and 40 ms used for calculating ${\mathrm{T}}_{2}^{*}$-maps. **c** Calculated ${\mathrm{T}}_{2}^{*}$-maps at three different time points during the dynamic BOLD measurement, marked with arrows on the acquisition timeline. **d** Regions of interest (ROIs) drawn on a mean image of the tree first echoes. The blue ROI is drawn in the gastrocnemius muscle (lateral and medial head) and the red ROI is drawn in the soleus muscle. **e** Mean ${\mathrm{T}}_{2}^{*}$-time curves of the two outlined muscles for the 5-min (left) and 1.5-min (right) occlusion duration

All subjects were examined with MRI lying down in a feet first, supine position. The examinations were performed on the right leg, positioned close to the center of the magnet bore for maximum field homogeneity. The left leg was positioned as far from the isocenter as possible, while maintaining a comfortable pose, to minimize susceptibility effects in the MR images. A wide leg tourniquet cuff (ERKA, Berlin, Germany, size: 34–43 cm) was wrapped around the thigh of the examined leg, as illustrated in Fig. 1a. To reduce the load on the calf from the table, the knee and the foot were well supported.

Before the MRI examination and the first cuff compression, the participants rested for at least 10 min on the MRI table. To assure full occlusion of the arteries to the lower leg, the cuff was inflated to a pressure ≥ 50 mmHg above the systolic arm pressure. The cuff inflation and deflation were achieved using an automatic tourniquet system (ATS 750, Zimmer, USA) which was placed in the control room and connected to the cuff via the wave trap. Further, the cuff inflation and deflation were initiated manually via the ATS by an assisting scanner operator and were achieved within 5 and 2 s, respectively. As illustrated in Fig. 1a, the cuff compression paradigm started with a 1-min baseline followed by cuff inflation and the 1.5- or 5-min ischemic period, after which cuff deflation and a 5-min post-occlusion period followed. During the entire cuff compression paradigm, ${\mathrm{T}}_{2}^{*}$-weighted MR images were acquired. Further details regarding MR imaging are found below.

### Study protocol

To study the difference in BOLD response for different occlusion durations, each participant was subject to the 5- and the 1.5-min occlusion cuff compression paradigms, acquired at two separate imaging sessions separated by at least 1 week.

To study intra-session repeatability, 11 of the 14 subjects went through the same cuff compression paradigm twice in both sessions, with the repeated measurement performed 10 min after the first. However, one of the repeated scans with 5-min occlusion had to be excluded since it was incompletely recorded due to an unwanted scanner pause during the dynamic measurement.

To study inter-session repeatability of the 5-min occlusion, six of the participants were asked to return for a third scanning session to repeat the 5-min cuff compression measurement. The repeated 5-min occlusion scanning session was more than 2 weeks after the first (mean ± SD = 24 ± 5 days).

Table 1 summarizes the cuff compression paradigms and the number of subjects included in each measurement. All subjects except for two were measured with the sessions in chronological order. One subject had Session 2 prior to Session 1 and one had Session 3 prior to Session 2.

**Table 1:** Resulting number of included subjects, *n*, for each measurement

	Measurement 1	Rest	Measurement 2
Session 1	5 min occlusion duration, S1M1, *n* = 14	10 min	5 min occlusion duration, S1M2, *n* = 10
Session 2	1.5 min occlusion duration, S2M1, *n* = 14	10 min	1.5 min occlusion duration, S2M2, *n* = 11
Session 3	5 min occlusion duration, S3M1, *n* = 6	–	–

Each sub-measurement denoted as SXMY, with *X* denoting the scan session and *Y* the measurement numbers. Occlusion duration, either 1.5 or 5 min, denoting which compression paradigm, was used for each scan session

### MR imaging

The BOLD MRI was performed on a clinical 3 T MRI scanner (Magnetom Skyra, Siemens Healthineers, Erlangen, Germany) using a large 4-channel receiver flex coil that was wrapped loosely around the calf. ${\mathrm{T}}_{2}^{*}$-weighted images (Fig. 1b) were acquired with a multi-echo fast low angle shot (FLASH) imaging sequence with 11 equidistant echoes at the echo times (TE): 2.0, 5.8, 9.6, 13.4, 17.2, 21.0, 24.8, 28.6, 32.4, 36.2, 40.0 ms, repetition time (TR): 44 ms, flip angle: 9 degrees, slice thickness: 10 mm, field of view: 160 × 160 mm^2^, acquisition matrix: 128 × 119 and parallel imaging acceleration factor (PAT): 2. The temporal resolution of the resulting ${\mathrm{T}}_{2}^{*}$-maps (Fig. 1c) were 3.2 s and a total of 207 or 141 consecutive ${\mathrm{T}}_{2}^{*}$-maps were produced for the long and the short cuff compression paradigm, respectively. Image acquisition was performed consecutively during the entire cuff compression paradigm. The cuff inflation and deflation co-occurred with the 19th and the 114th/47th (long/short cuff compression paradigm) imaging frame, respectively.

### Image post-processing

Post-processing and semi-quantitative analyses of all images was performed using a dedicated analysis software for calf muscle perfusion initially developed by Antaros Medical and further customized in-house. The software was written in Python [31] (Python Software Foundation. Python Language Reference, version 3.7.5.), Cython [32], and with graphical user interfaces provided via Jupyter Notebooks [33]. Following data import, ${\mathrm{T}}_{2}^{*}$-estimates were produced voxel wise to form ${\mathrm{T}}_{2}^{*}$-maps over the entire cuff compression paradigm with a temporal resolution of 3.2 s. ${\mathrm{T}}_{2}^{*}$-maps were computed based on a pixel-wise log-linear regression applied to the ${\mathrm{T}}_{2}^{*}$-weighted images from the 11 echoes. Manual regions of interest (ROIs) were outlined by one out of two observers (JA and SE) in the first ${\mathrm{T}}_{2}^{*}$-weighted image using SmartPaint [34], example ROIs shown in Fig. 1d. ROIs for S1M1 in all included subjects (*n* = 14) were outlined by both observers and one observer (JA) did the outlining twice, 3 weeks apart. To address spatio-temporal subject movement, the ROIs were transformed to each data frame in the time series using an atlas-based segmentation scheme, where 2D non-rigid registrations were carried out using the software Elastix [35]. Finally, ROI-wise ${\mathrm{T}}_{2}^{*}$-time curves (Fig. 1e) were extracted by averaging the voxel-wise estimates within each ROI for each time point.

### Semi-quantitative ${\mathrm{T}}_{2}^{*}$-time curve analysis

**Fig. 2:**
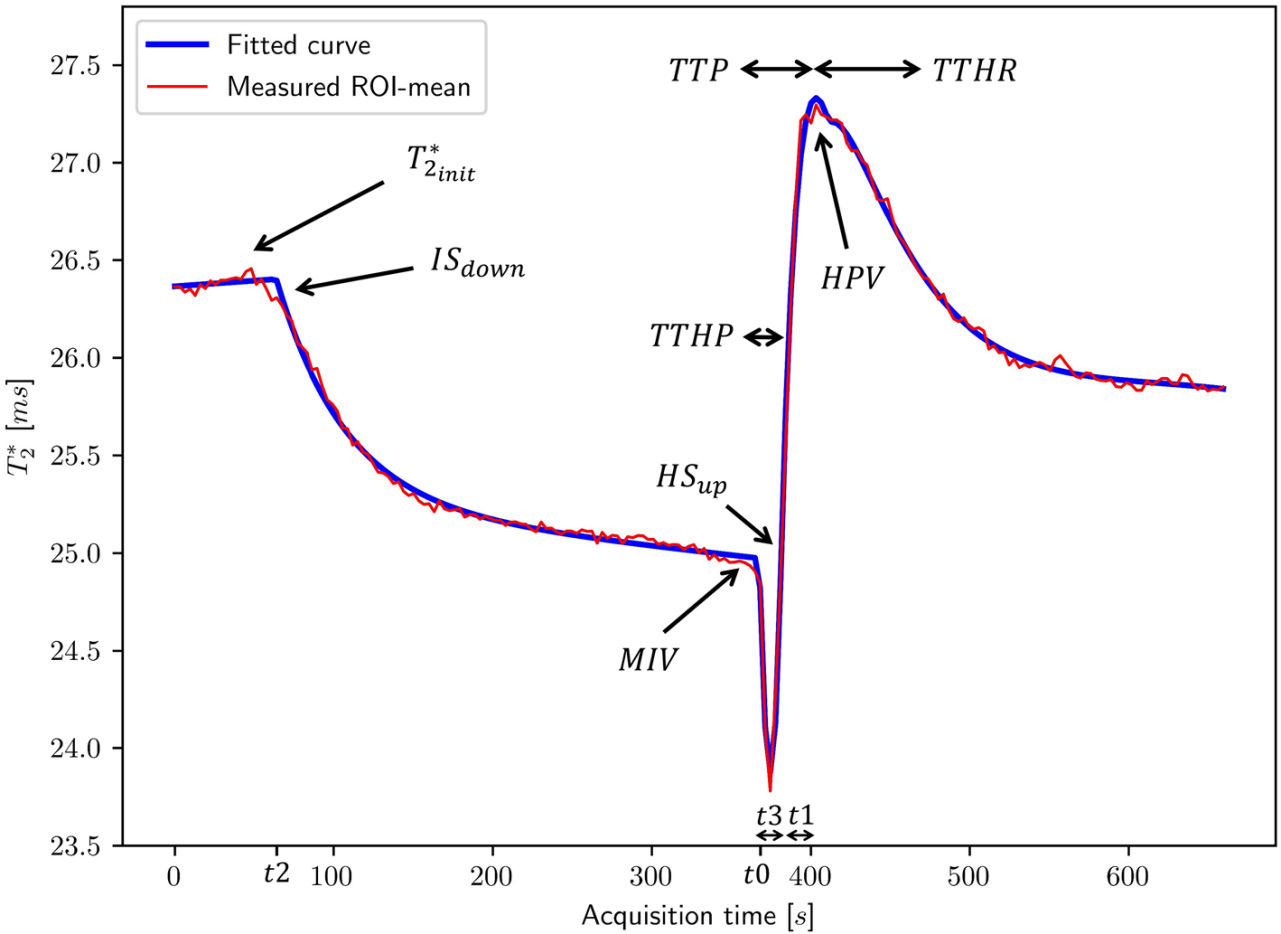
The descriptive model (blue) fitted to the ROI-mean ${\mathrm{T}}_{2}^{*}$-data (red) and the temporal location of derived semi-quantitative parameters: initial ${\mathrm{T}}_{2}^{*}$-value (${{\mathrm{T}}_{2}^{*}}_{\mathrm{init}}$), initial declining slope (${\mathrm{IS }}_{\mathrm{down}}$), minimum ischemic value (MIV), hyperemic upslope (${\mathrm{HS }}_{\mathrm{up}})$, time to reach half of the reactive hyperemic peak value (TTHP), hyperemic peak value (HPV), time to reactive hyperemic peak (TTP), and time to half recovery (TTHR), illustrated with arrows (levels and slopes) and double-headed arrows (time and level intervals). Time points t0–t3 define the borders for the different segments of the descriptive model, and a detailed description is found in Appendix

A semi-automatic parameter analysis was carried out by first fitting a parameterized function to a baseline-normalized ${\mathrm{T}}_{2}^{*}$-time curve. Most curves were fitted with default upper/lower limits and start values of the model input parameters that suited most curves. However, for some of the curves custom limits were set manually by the observer to improve the fit. There was always only one set of limits and start values used for each measurement, i.e*.*, the same values were used for both gastrocnemius and soleus. A more detailed description of the fitted descriptive model is found in Appendix. Figure 2 shows a typical time curve from a 5-min occlusion measurement together with the temporal location of the derived BOLD parameters. The derived semi-quantitative parameters are the initial ${\mathrm{T}}_{2}^{*}$-value (${{\mathrm{T}}_{2}^{*}}_{\mathrm{init}}$), initial declining slope (${\mathrm{IS}}_{\mathrm{down}}$), minimum ischemic value (MIV), hyperemic upslope (${\mathrm{HS}}_{\mathrm{up}})$, time to reach half of the reactive hyperemic peak value (TTHP), hyperemic peak value (HPV), time to reactive hyperemic peak (TTP), and time to half recovery (TTHR). Semi-quantitative parameters are derived in Appendix.

### Statistical analysis

All continuous data are presented as median and inter quartile range if not otherwise stated. Statistical evaluations were performed in the statistical scripting language R [36]. No Bonferroni corrections were performed.

The non-parametric paired Wilcoxon Signed-Rank test with a 95% confidence interval was used to evaluate differences between inter- and intra-session measurements for the two ROI positions.

Subject-wise coefficient of variation, ${\mathrm{CV}}_{\mathrm{w}}$, was used to evaluate inter- and intra-session reproducibility. Inter-session reproducibility was determined from the six participants that were measured on a third occasion with the longer 5-min cuff compression paradigm.

The single score intraclass correlation (ICC(2,1)) with two operators was used to evaluate derived parameters in regard to inter- and intra-operator reproducibility of manual steps in the data post-processing chain [37]. ICC values were interpreted in accordance with Koo and Li [37], based on the lower 95% confidence interval as poor (ICC < 0.5), moderate (0.50 ≤ ICC ≤ 0.75), good (0.75 ≤ ICC ≤ 0.9), and excellent (ICC > 0.9).

## Results

**Fig. 3:**
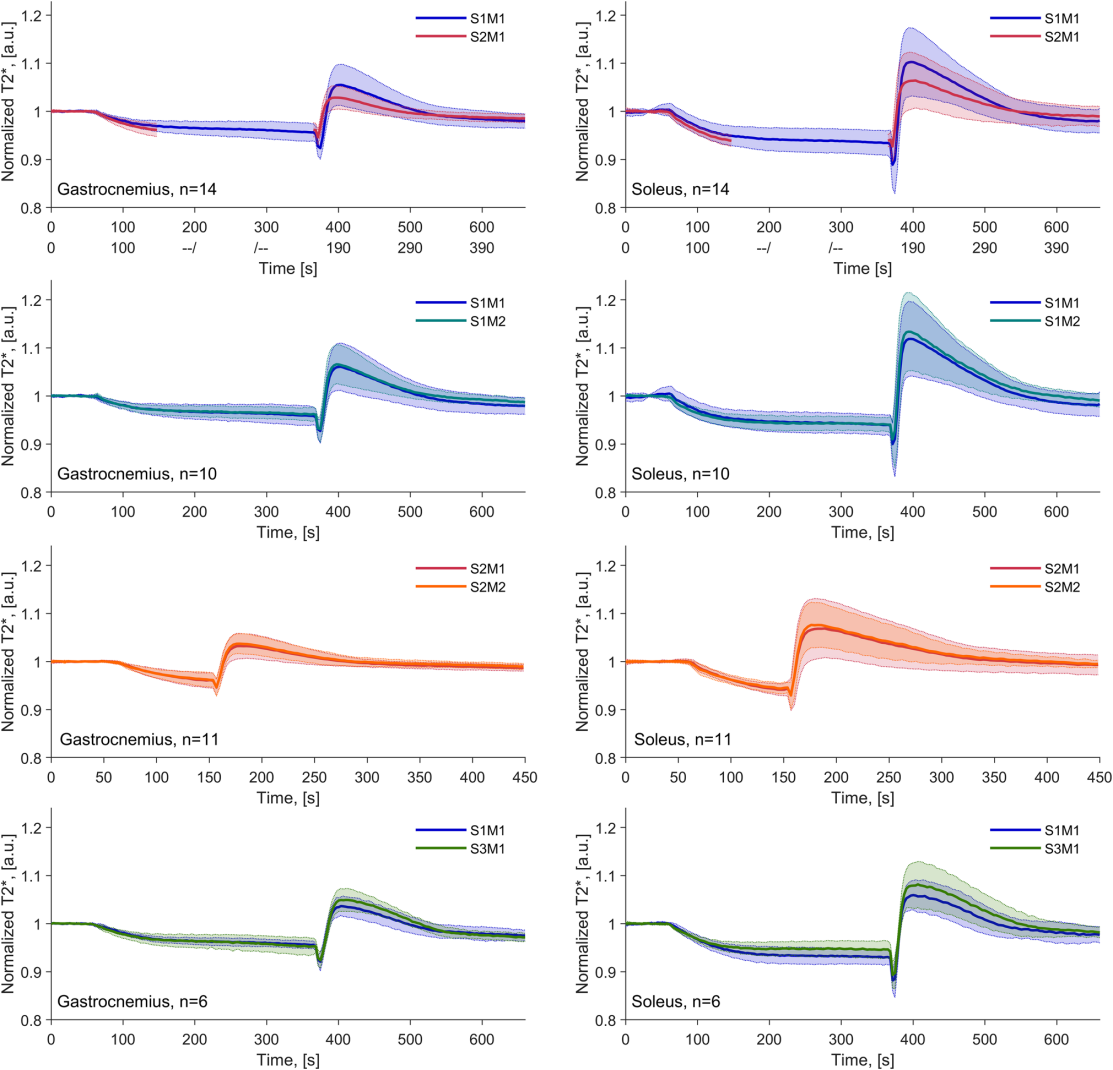
Baseline-normalized ${\mathrm{T}}_{2}^{*}$-time curves for compared datasets, displaying the subject mean time curves as filled lines and the subject standard deviation as transparent area and dotted lines. The gastrocnemius muscles are displayed to the left and soleus muscles to the right. The top row shows, long and short occlusion durations plotted together with the second half of the short-duration curve shifted so that the timings of deflation coincide. Second row shows two repeated 5-min cuff compression paradigms. Timings between the first deflation and the second inflation is approximately 10 min. Third row shows two repeated one-and-a half-minute cuff compression paradigms. The time between the first deflation and the second inflation was approximately 10 min. The bottom row shows the inter-session evaluation with a 5-min cuff compression paradigm scanned at least 2 weeks apart

Subject mean and standard deviation plots of normalized ${\mathrm{T}}_{2}^{*}$-time curves are displayed in Fig. 3. The top row displays the BOLD curves for the 5-min (S1M1) and 1.5-min (S2M1) cuff compression paradigms. Note that the longer arterial occlusion appears to produce BOLD curves with a higher hyperemic peak and increased variability between subjects at time points close to the peak. Overall, the intra-session curves in the second (5-min occlusion) and third row (1.5-min occlusion) show high levels of agreement in both mean and variability up until cuff deflation. After cuff release, the peak of the second measurement (SXM2) appears higher than that of the first measurement (SXM1) for both muscles, however, slightly more so in gastrocnemius. Inter-scan measurements shown in the fourth row display some discrepancies in amplitude. Firstly, the ischemic period for the second measurement (S3M1) in the soleus ROI reaches a lower ischemic level than for the first measurement (S1M1). Secondly, after cuff release, for both muscle types, the mean time curve of the later measurement remains higher than that of the first, throughout the remaining duration of the measurement. Corresponding curves from the inter- and intra-observer evaluations are included in Sup. Fig. S8 and display high levels of agreement over the entire measurement.

### Differences between long and short occlusion duration

**Fig. 4:**
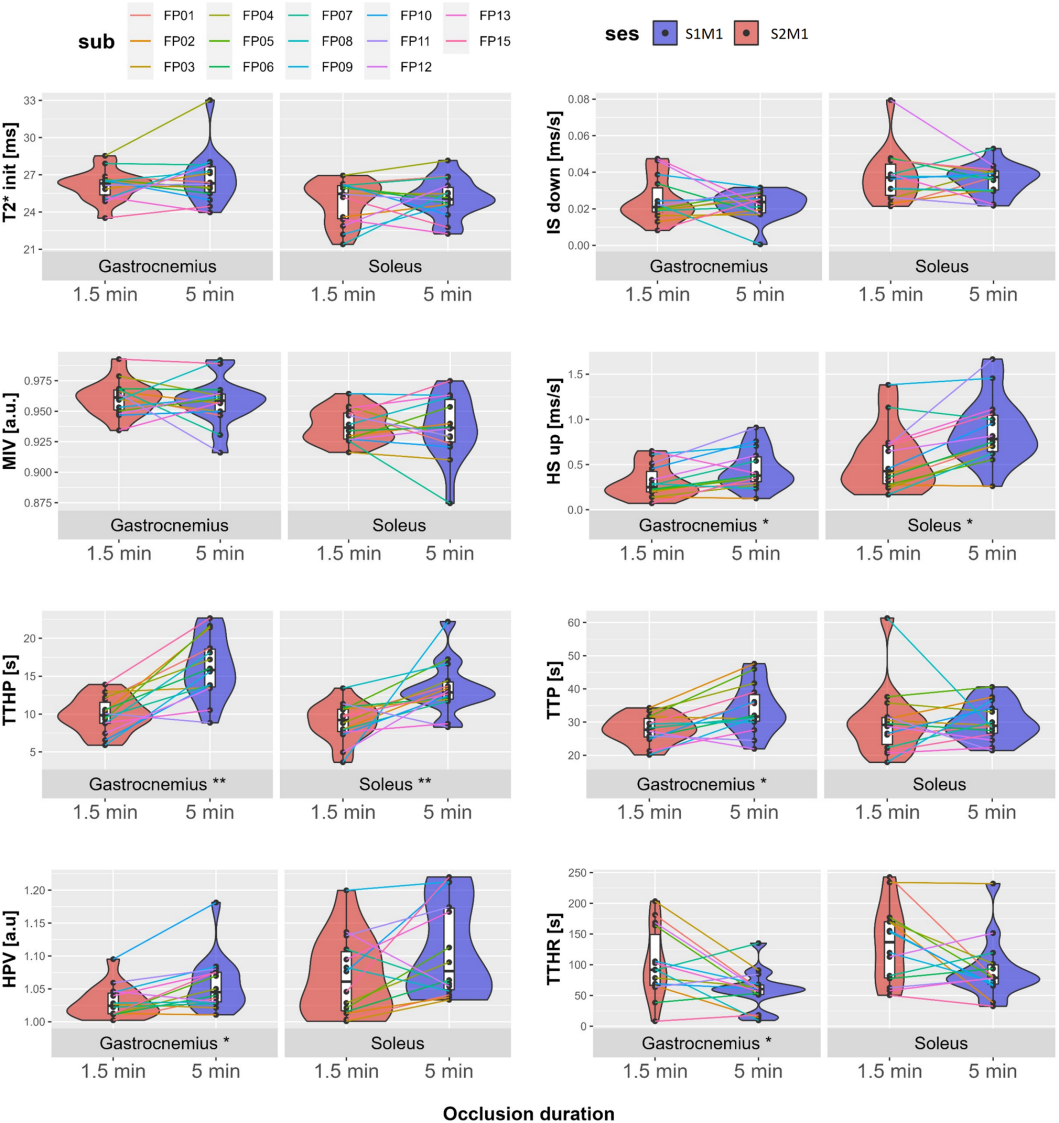
Pair-wise parameter levels and group-wise distributions (violin plots) from the long (5 min) and short (1.5 min) occlusion duration (*n* = 14), evaluated for semi-quantitative parameters initial ${\mathrm{T}}_{2}^{*}$-value (${{\mathrm{T}}_{2}^{*}}_{\mathrm{init}}$), initial declining slope (${\mathrm{IS }}_{\mathrm{down}}$), minimum ischemic value (MIV), hyperemic upslope (${\mathrm{HS }}_{\mathrm{up}})$, time to reach half of the reactive hyperemic peak value (TTHP), hyperemic peak value (HPV), time to reactive hyperemic peak (TTP) and time to half recovery (TTHR). Significance at *p* < 0.05 and *p* < 0.005 is indicated with a * and ** besides the ROI name for each subset

The effect of 5- and 1.5-min occlusion on the semi-quantitative parameters is shown in Table 2. Parameter changes for each individual are displayed in violin plots in Fig. 4. No significant differences were found for the parameters describing the curve before cuff deflation, ${{\mathrm{T}}_{2}^{*}}_{\mathrm{init}}, {\mathrm{IS}}_{\mathrm{down}},$ or MIV (*p* > 0.05). However, all parameters describing the curve after cuff deflation showed significant differences between short and long occlusion duration in gastrocnemius (*p* < 0.05). Significant differences in both muscles were found for ${\mathrm{HS}}_{up}$ (*p* < 0.05) as well as for TTHP that showed the most significant difference (*p* < 0.0003). Bland–Altman plots, seen in Sup. Fig. S1, show biases between measurements with different occlusion durations in all hyperemic parameters except for TTP in soleus.

**Table 2:** Median ($\widetilde{{\varvec{x}}}$) and inter quartile range (IQR) for the semi-quantitative parameters from the 14 subjects for the two different cuff compression paradigms with 5- and 1.5-min occlusion

Parameter	Muscle	Long 5 min $\widetilde{x}$ (IQR)	Short 1.5 min $\widetilde{x}$ (IQR)	$\Delta \widetilde{x}$ (%)	*p*-Value
${{\mathrm{T}}_{2}^{*}}_{\mathrm{init}}$(ms)	Gastrocnemius	26.3 (2.1)	26.3 (1.2)	0.0	0.7
	Soleus	25.1 (1.4)	25.5 (2.7)	1.6	0.9
${\mathrm{IS }}_{\mathrm{down}}$*10^2^ (ms/s)	Gastrocnemius	2.4 (0.92)	2.1 (1.3)	13.3	0.9
	Soleus	3.7 (1.0)	3.7 (1.5)	0.0	0.98
MIV (a.u.)	Gastrocnemius	0.96 (0.01)	0.96 (0.02)	0.0	0.5
	Soleus	0.94 (0.04)	0.94 (0.02)	0.0	0.98
${\mathrm{HS}}_{up}$*10^2^ (ms/s)	Gastrocnemius	37.9 (27.6)	25.0 (22.6)	41.0	** < 0.05**
	Soleus	78.3 (40.2)	42.3 (42.2)	59.7	**0.03**
TTHP (s)	Gastrocnemius	15.8 (5.0)	9.8 (2.8)	46.9	**8e-05**
	Soleus	12.9 (2.3)	9.2 (2.9)	33.5	**0.0003**
TTP (s)	Gastrocnemius	31.7 (8.0)	27.7 (5.6)	13.5	**0.02**
	Soleus	28.8 (7.25)	29.1 (8.1)	1.0	0.7
HPV (a.u.)	Gastrocnemius	1.04 (0.05)	1.02 (0.03)	1.9	**0.04**
	Soleus	1.08 (0.12)	1.06 (0.09)	1.9	0.1
TTHR (s)	Gastrocnemius	60.4 (15.3)	91.7 (82.6)	41.2	**0.01**
	Soleus	78.8 (31.2)	136.5 (91.2)	53.6	0.08

$\Delta \widetilde{\mathbf{x}}$ denotes the percentage difference in median parameter values between 1.5- and 5-min occlusion durations. Statistically significant differences are written in bold digits

*IQR* inter quartile range; ${{T}_{2}^{*}}_{init}$ Initial ${\mathrm{T}}_{2}^{*}$; ${IS }_{down}$ Initial downslope; *MIV* minimal ischemic value; ${HS}_{up}$ hyperemic upslope; *TTHP* time to hyperemic peak; *TTP* time to peak; *HPV* hyperemic peak value; *TTHR* time to half recovery

### Repeatability

**Fig. 5:**
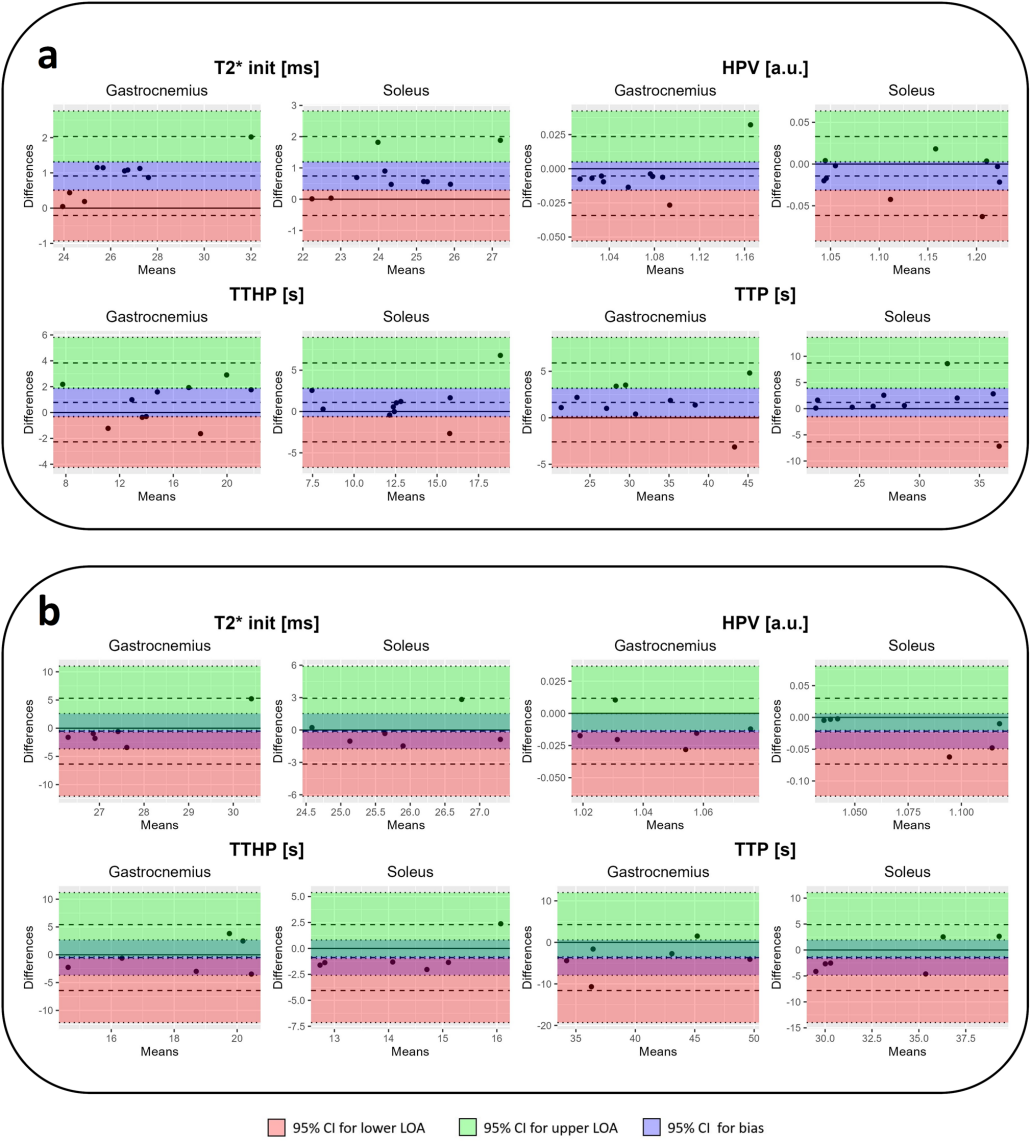
Bland–Altman style plots for intra-session (**a**) and inter-session (**b**) of the 5-min occlusion paradigm repeatability evaluations covering a selection of semi-quantitative parameters including initial ${\mathrm{T}}_{2}^{*}$-value (${{\mathrm{T}}_{2}^{*}}_{\mathrm{init}}$), hyperemic peak value (HPV), time to reach half of the reactive hyperemic peak value (TTHP) and time to reactive hyperemic peak (TTP). The 95% confidence interval for the lower and upper limits of agreement as well as the bias are shaded in red, green, and blue, respectively

Figure 5 shows Bland–Altman style plots for a selection of parameters covering intra- and inter-session evaluations with 5-min cuffing. The second measurement in the intra-session measurements (Fig. 5a and Sup. Figs. S2–S5) yielded higher ${\mathrm{T}}_{{2}_{init}}^{*}$ values for both the 1.5- and 5-min occlusions. Also, for gastrocnemius, the bias for the parameter TTP seen in Fig. 5a and from the measurement data in Sup. Fig. S2 indicates lower TTP values for the second intra-session measurement. Individual parameter changes for inter-session measurements can be seen in Sup. Figs. S6 and S7. In the corresponding Bland–Altman plot (Sup. Fig. S7), there appears to be a bias toward higher HPV values in the second measurement.

Table 3 presents estimates of CV_w_ for the semi-quantitative parameters covering intra-session repeatability with 5- and 1.5-min occlusions as well as inter-session repeatability for measurements with 5-min occlusion. ICC estimates for inter- and intra-subject reproducibility are shown in the two right-most columns. Independent on muscle type, the ICC was excellent for both inter- and intra-operator repeatability for all parameters, except for TTP and TTHP in Soleus. The violin plots in Sup. Figs. S9 and S11 as well as Bland–Altman plots in Sup. Figs. S10 and S12 support these results and show a high level of agreement between observations for most parameters.

**Table 3:** ${\mathrm{CV}}_{\mathrm{w}}$ for all repeatability evaluations, inter- and intra-session for gastrocnemius and soleus, and for the long or short occlusion duration

Parameter	Muscle	Intra-session ${\mathrm{CV}}_{w}$ (%) long *n* = 10	Intra-session ${\mathrm{CV}}_{w}$ (%) short *n* = 11	Inter-session ${\mathrm{CV}}_{w}$ (%) long *n* = 6	Intra-operator $ICC$ long *n* = 14	Inter-operator $ICC$ long *n* = 14
${{\mathrm{T}}_{2}^{*}}_{\mathrm{init}}$	Gastrocnemius	2.7	1.7	6.7	0.99	0.999
	Soleus	2.7	1.7	3.8	0.99	0.99
${\mathrm{IS}}_{\mathrm{down}}$	Gastrocnemius	45.5	17.4	24.7	0.96	0.959
	Soleus	26.0	11.76	21.2	0.96	0.399
MIV	Gastrocnemius	0.9	0.4	1.5	0.99	0.99
	Soleus	1.1	0.7	2.0	0.99	0.93
${\mathrm{HS}}_{\mathrm{up}}$	Gastrocnemius	17.6	10.15	15.3	0.99	0.92
	Soleus	13.7	20.6	12.1	0.85	0.99
TTHP	Gastrocnemius	8.9	16.5	10.5	0.99	0.82
	Soleus	12.4	25.0	8.4	0.99	0.01
TTP	Gastrocnemius	5.6	8.6	9.9	0.82	0.86
	Soleus	8.2	10.8	7.1	0.92	0.44
HPV	Gastrocnemius	0.9	0.7	1.2	0.99	0.99
	Soleus	1.6	1.8	2.0	0.99	0.99
TTHR	Gastrocnemius	37.8	32.7	34.3	0.93	0.95
	Soleus	22.5	21.0	17.7	0.99	0.92

The lower 95% percentile for the ICC estimate is shown for inter- and intra-operator evaluation for the long occlusion duration

${CV}_{w}$ coefficient of variation; $ICC$ intraclass correlation, ${{T}_{2}^{*}}_{init}$ initial ${\mathrm{T}}_{2}^{*}$; ${IS}_{down}$ initial downslope; $MIV$ minimal ischemic value; ${HS}_{up}$ hyperemic upslope; $TTHP$ time to half peak; $TTP$ time to peak; $HPV$ hyperemic peak value; $TTHR$ time to half recovery

## Discussion

The present study shows that the occlusion duration influences the hyperemic response with an increased hyperemic BOLD response for longer arterial occlusion durations. The occlusion duration was found to influence semi-quantitative BOLD parameters in gastrocnemius with high inter- and intra-session repeatability. Therefore, this study highlights that the cuffing duration should be considered in the development of consensus protocols for the further development of cuff-based dynamic BOLD imaging.

### Differences in BOLD parameters between long and short ischemic duration

In parameters associated with the hyperemic response, HPV, ${\mathrm{HS}}_{\mathrm{up}}$, TTHP, TTP, and TTHR were found to be sensitive to the occlusion duration. For TTHP and ${\mathrm{HS}}_{\mathrm{up}}$, significant differences were found in both muscles, while HPV, TTP, and TTHR were found significant only for gastrocnemius. However, the Bland–Altman plots covering the occlusion duration evaluation (Sup. Fig. S1) indicate a bias for all hyperemic parameters except for TTP in soleus.

Parameters ${\mathrm{HS}}_{\mathrm{up}}$ and TTHP, that describe the upslope during reactive hyperemia, yielded the most significant differences between occlusion durations. These parameters also gave higher levels of CV_w_ (CV_w_ > 20 and 25%, respectively) when decreasing the cuffing duration from a 5- to 1.5-min cuffing, compared to other parameters. Arguably these parameters may be the most difficult to reproduce as they describe the shortest part of the curve. Even so, both the inter- and intra-session CV_w_ remained lower than the differences in these parameters found when comparing occlusion durations. So, while repeatability may be high in comparison, these parameters also show a higher sensitivity to physiological change, which we expect to be useful when studying a more diverse subject group with varying degrees of pathophysiology.

The increase in variability as the occlusion duration decreases may reflect differences in the subjects regarding both the level of reached muscle ischemia and the tissue’s ability to re-oxygenate. By using a longer occlusion, we reason that to a larger extent, subjects will have reached sufficient ischemia and that parameters describing the hyperemic response will better isolate tissue re-oxygenation effects.

Most previous studies have implemented a 3–5-min arterial occlusion. The 1.5-min occlusion for the comparison acquisition in this study was selected empirically based on when most subjects begin reaching the plateau in the ischemic phase of the ${\mathrm{T}}_{2}^{*}$-time curve. We expect that an evaluation of differences in derived hyperemic parameters between 3- and 5-min cuffing durations would be smaller than what has been found in this study.

Evaluating the differences in derived parameters between long and short occlusion duration is relevant for gaining a better understanding of the underlying physiology as well as for the methodology of the technique. In respect to physiology, the increased hyperemic response for the longer occlusion indicated that the occlusion duration indeed influences the calf muscle re-oxygenation. In respect to methodology, these findings translate to an increase in dynamic range for several of the derived BOLD parameters. This may further improve the techniques sensitivity to distinguish between levels of vascular dysfunction. Looking ahead, we expect the occlusion duration to be considered as a design parameter when establishing recommendations for BOLD imaging protocols of skeletal muscle.

### Measurement precision and clinical application

Regarding inter- and intra-session repeatability, as seen in Table 3, our results appear similar to previous results for 5-min occlusion durations in the literature. TTP and HPV have been evaluated with similar levels of repeatability of 1.1% and 15.3% intra-session CV_w_ and 2.0% and 16.5% inter-session CV_w,_ for TTP and HPV [7].

In our intra-session evaluation, all individuals showed a lower ${T}_{{2}_{\mathrm{init}}}^{*}$ in the second measurement indicating that a 10-min rest between cuff compressions was not sufficient for the muscles to return to baseline oxygenation level. Interestingly, when observing Bland–Altman plots of intra-session repeated measurements in Fig. 5a, other parameters also seemed to be affected in the repeated measurement, specifically, TTP, which was lower in the repeated 5-min occlusion measurement for all individuals but one. This indicates that the first measurement introduces physiological change in the skeletal muscle that is detected by the BOLD measurement. In clinical settings, re-measurements can occur due to several reasons, awareness of such effects, and the effect on pathophysiology with this technique may be important in clinical practice.

The low number of data points in the inter-session evaluation, seen in the Bland–Altman plots of Fig. 5b, provides limited possibilities to draw conclusions. However, as we compare ${T}_{{2}_{\mathrm{init}}}^{*}$ with that of the intra-session evaluation in Fig. 5a, we see no bias, as expected. Further, in accordance with the increase in hyperemic response seen in the bottom row of Fig. 3, there appears to be a bias in HPV. Possibly, these observations can be explained by actual physiological differences. The participating subjects were not asked to avoid caffeine or other drugs before examinations. Caffeine and antihistamines have been shown to affect the BOLD signal [38] and so variabilities in caffeine consumption between the different days of scanning could be a source of variation. Further, almost 5 weeks separated the two measurements in time for one of the subjects. During such a long timeframe, the muscle tissue may have gone through physiological change. Other sources of inter-session variability include imaging-related factors, such as the positioning of the legs inside the magnet, image slice position, and differences in the application of the cuff, which may have varied slightly between the sessions.

The parameters ${\mathrm{IS}}_{\mathrm{down}}$, MIV, ${\mathrm{HS}}_{\mathrm{up}},$ and TTHR yielded slightly improved intra-session repeatability for the 1.5-min paradigm when compared to the 5-min cuff compression paradigm. But in general, the observed differences in CV_w_ between occlusion durations were small and with the small sample size in mind, should be regarded negligible.

The inter- and intra-session repeatability evaluations provide an insight into the measurement precision of the technique. By comparing the intra- and inter-session variability of healthy subjects presented herein with previously published differences in parameter levels between healthy subjects and patient groups, we can assess the clinical relevance of the technique’s precision. A previous publication has shown that the CV_w_ of patients with PAD in general was 10% larger when compared to healthy subjects and that differences between these groups for parameters HPV and TTP was as large as ~ 5% and 143%, respectively [7]. In another study based on patients with CLI, parameters corresponding to ${\mathrm{HS}}_{up}$ and MIV display differences between healthy and ischemic limbs as large as 59% and 27%, respectively [10]. As displayed in Table 3, inter-session CV_w_ for parameters HPV, TTP, ${\mathrm{HS }}_{\mathrm{up}},$ and MIV were ~ 1–2%, ~ 7–10%, ~ 12–15, and ~ 1.5–2%, respectively. This indicates that differentiation of disease severity may be feasible with the technique given the current inter- and intra-scan variability.

In general, variabilities related to manual post-processing steps of the imaging procedure in this study were found to be small, indicating that the technique, in this regard, may be suitable for clinical settings. The inter- and intra-observer repeatability evaluation yielded excellent results in terms of ICC, and the summary time curves in Sup. Fig. S8 were almost perfectly overlapped. In the inter-observer evaluation, TTP and TTHP stood out with low ICC. When studying the underlying raw data (not shown), the cause was found to be due to the fitting of one outlier curve, where one observer defined the fitting range for the cuff release time point incorrectly, an error which easily could have been avoided by enforcing a hard limit to the ranges of this parameter in the software. With this in mind, we believe that the driving factors to help achieve a low operator dependency include the use of clearly defined operator guidelines for the semi-automatic curve fitting procedure and ROI delineation, as well as the use of automated motion correction software during the data pre-processing.

### Limitations

The present study has some limitations. For the estimation of the ICC estimates, at least 30 heterogeneous samples and three observers should be used [37]. In this work only two operators and 14 scans were used for the ICC estimates, and as such, ICC estimates may have been inflicted by individual extreme value data points. Similarly, the same issue with a limited number of data points is relevant for the CV_w_ estimates used in the inter- and intra-session reproducibility evaluations. The small number of data points in these evaluations, *n* = 10, 11, and 6, increases the sensitivity of the CV_w_ estimate to individual deviating estimates.

## Conclusion

This study shows that semi-quantitative parameters in skeletal muscle BOLD perfusion measurements are indeed sensitive to the occlusion duration. The longer 5-min occlusion produced an increased hyperemic signal effect compared to the shorter 1.5-min occlusion. Derived parameters showed statistically significant differences (*p* < 0.05) in gastrocnemius for all parameters describing the hyperemic response and in soleus for two of these parameters. The relatively small intra- and inter-session variability in derived parameters that describe the hyperemic response indicate that the technique continues to show potential as a tool for differentiation of vascular dysfunction in peripheral skeletal muscle. Finally, most derived parameters demonstrated an excellent inter- and intra-observer repeatability, indicating that the technique is robust and can offer a diagnostic tool that seems to be independent on the observer interaction.

### Electronic supplementary material

Below is the link to the electronic supplementary material.

Supplementary file1 Supporting Figures. S1–S7 and S9–S12 display violin and Bland–Altman plots of all derived semi-quantitative BOLD parameters for comparisons between long and short occlusion durations, intra- and inter-session repeatability evaluation (intra-session for both 1.5- and 5-minute occlusions), as well as intra- and inter-operator evaluations. Sup. Fig. S8 displays summary BOLD time curves for inter- and intra-observer evaluations. (PDF 2777 KB)

## Data Availability

Not applicable.

## Descriptive BOLD ${\mathrm{T}}_{2}^{*}$-time curve model

The descriptive data representation was carried out by first fitting a parameterized function, $f\left(t,\theta \right)$, to the ${\mathrm{T}}_{2}^{*}$-time curve. This signal representation was based on the assumptions that the ${\mathrm{T}}_{2}^{*}$ will decrease during ischemia and increase rapidly to an overshoot during reactive hyperemia, followed by a slow decline to a baseline level. The function was developed empirically to be flexible enough to adhere to the measured data while keeping down the total number of fitting parameters. The function consists of several components defined over specified time intervals according to

$$f\left(t,\theta \right)=m\left(t\right)+n\left(t\right)+u\left(t\right)+g\left(t\right)+s\left(t\right)+l(t)$$

and where

$$m\left(t\right)=1+{m}_{1}\left(t-\frac{{t}_{2}}{2}\right),\,t\le {t}_{2}$$

$$n\left(t\right)=m\left({t}_{2}\right)-{n}_{0}\left(1-{e}^{1-{n}_{1}\left(t-{t}_{2}\right)}\right),\,t\ge {t}_{2}, \, t\le {t}_{0}$$

$$u\left(t\right)=n\left({t}_{0}\right)+{u}_{0}t\left(t-{t}_{3}\right),t\ge {t}_{0}, \, t\le {t}_{0}+{t}_{3}$$

$$g\left(t\right)={g}_{0}\left(1-{e}^{-{g}_{1}\left(t-{t}_{0}\right)}\right){e}^{-{g}_{2}\left(t-{t}_{0}\right)},\,t>{t}_{0}+{t}_{3}$$

$$s\left(t\right)={s}_{0}\left(1-{e}^{-{s}_{1}\left(t-{t}_{0}-{t}_{1}\right)}\right),\,t>{t}_{0}+{t}_{1}+{t}_{3}$$

$$l\left(t\right)=n\left({t}_{0}\right)+{l}_{1}\left(t-{t}_{0}\right),\,t >{t}_{0}+{t}_{3}$$

The expressions for *g*(*t*) and *s*(*t*), is a development of the gamma variate and sigmoidal function components published previously by Schewsow et al. [39] modified so that the derivative at the hyperemic upslope is defined. The linear function *l*(*t*) accounts for any signal drift during the hyperemic phase. The model parameters $\theta =[{m}_{1}, {n}_{0}, {n}_{1},{u}_{0},{g}_{0}$, ${g}_{1}$,${g}_{2}, {s}_{0}$,${s}_{1}$, ${l}_{1}$,${t}_{0}$,${t}_{1}$, ${t}_{2}, {t}_{3}]$ were fitted by minimizing the sum of squared errors between the fitted curve, $f\left(t,\theta \right)$, and the time curve. The time curve is the derived absolute ${\mathrm{T}}_{2}^{*}$-time curve normalized by the initial ${\mathrm{T}}_{2}^{*}$-level $({{T}_{2}^{*}}_{\mathrm{init}})$. The time intervals were dictated by the temporal landmarks t2 (cuff activation), t0 (cuff release), and t3 (duration of cuff release-induced undershoot). The time point, t1, dictates the delay for when the function segment *s*(*t*) is added to the hyperemic overshoot segment.

Except for ${{\mathrm{T}}_{2}^{*}}_{\mathrm{init}}$, all descriptive parameters were extracted from the fitted signal representation above. Table 4 describes the calculation of these parameters based on the descriptive function parameters.

**Table 4:** Semi-quantitative BOLD parameters and their estimation based on parameters from the fitted ${\mathrm{T}}_{2}^{*}$-time curve function

Parameter	Calculation	Description
t0	–	Cuff deflation time point (s)
t1	–	Delay from undershoot end to S(t) is added
t2	–	Cuff inflation time point (s)
t3	–	Undershoot duration (S)
${{\mathrm{T}}_{2}^{*}}_{\mathrm{init}}$	${{\mathrm{T}}_{2}^{*}}_{\mathrm{init}}=\frac{1}{{N}_{\mathrm{baseline}}}\sum_{{j}_{t}\in \mathrm{baseline}}\frac{1}{{N}_{\mathrm{ROI}}}\sum_{i\in \mathrm{ROI}}s(i,{j}_{t})$	Initial ${\mathrm{T}}_{2}^{*}$-level (ms)
Initial downslope (${\mathrm{IS}}_{\mathrm{down}}$)	${{\mathrm{T}}_{2}^{*}}_{\mathrm{init}}{n}_{0}{n}_{1}$	Curve slope after cuff inflation (ms/s)
$\mathrm{Minimum ischemic value }(\mathrm{MIV}$)	$(n\left({t}_{0}\right)-m\left({t}_{2}\right))$	Minimum ischemic value prior cuff release, relative to ${{\mathrm{T}}_{2}^{*}}_{\mathrm{init}}$ (a.u.)
Hyperemic peak value (HPV)	$\mathrm{max}(s(t))$	Hyperemic peak value, relative to ${{\mathrm{T}}_{2}^{*}}_{\mathrm{init}}$ (a.u.)
Time to half peak (TTHP)	${t}_{{\frac{\mathrm{HPV}}{2}}^{\mathrm{pre}}}-{t}_{0}$	Time from cuff deflation to reach half-way to the HPV (s), where ‘pre’ in the exponent indicates that this is a time point prior to $\mathrm{HPV}$
Time to peak (TTP)	${t}_{\mathrm{HPV}}-{t}_{0}$	Time from cuff deflation to HPV (s)
Hyperemic upslope (${\mathrm{HS}}_{\mathrm{up}})$	${{\mathrm{T}}_{2}^{*}}_{\mathrm{init}}({g}_{0}{g}_{1}+{l}_{1})$	Slope toward peak after cuff deflation and undershoot (s^−1^)
Time to half recovery (TTHR)	${t}_{{\frac{\mathrm{HPV}}{2}}^{\mathrm{post}}} -{t}_{\mathrm{HPV}}$	Time from $\mathrm{HPV}$ (s) to reach half-way to base line level, where ‘post’ in the exponent indicates that this is a time point after HPV
