# Supplementary material for: Arterial occlusion duration affects the cuff-induced hyperemic response in skeletal muscle BOLD perfusion imaging as shown in young healthy subjects
Source: MAGMA. 2023 Jun 17;36(6):897–910. doi: 10.1007/s10334-023-01105-y (PMC10667151; doi:10.1007/s10334-023-01105-y)
Supplement: Supplementary file 1 — Supplementary file1 Supporting Figures. S1–S7 and S9–S12 display violin and Bland–Altman plots of all derived semi-quantitative BOLD parameters for comparisons between long and short occlusion durations, intra- and inter-session repeatability evaluation (intra-session for both 1.5- and 5-minute occlusions), as well as intra- and inter-operator evaluations. Sup. Fig. S8 displays summary BOLD time curves for inter- and intra-observer evaluations. (PDF 2777 KB) [file 10334_2023_1105_MOESM1_ESM.pdf]

Supplementary materials

Occlusion duration evaluation

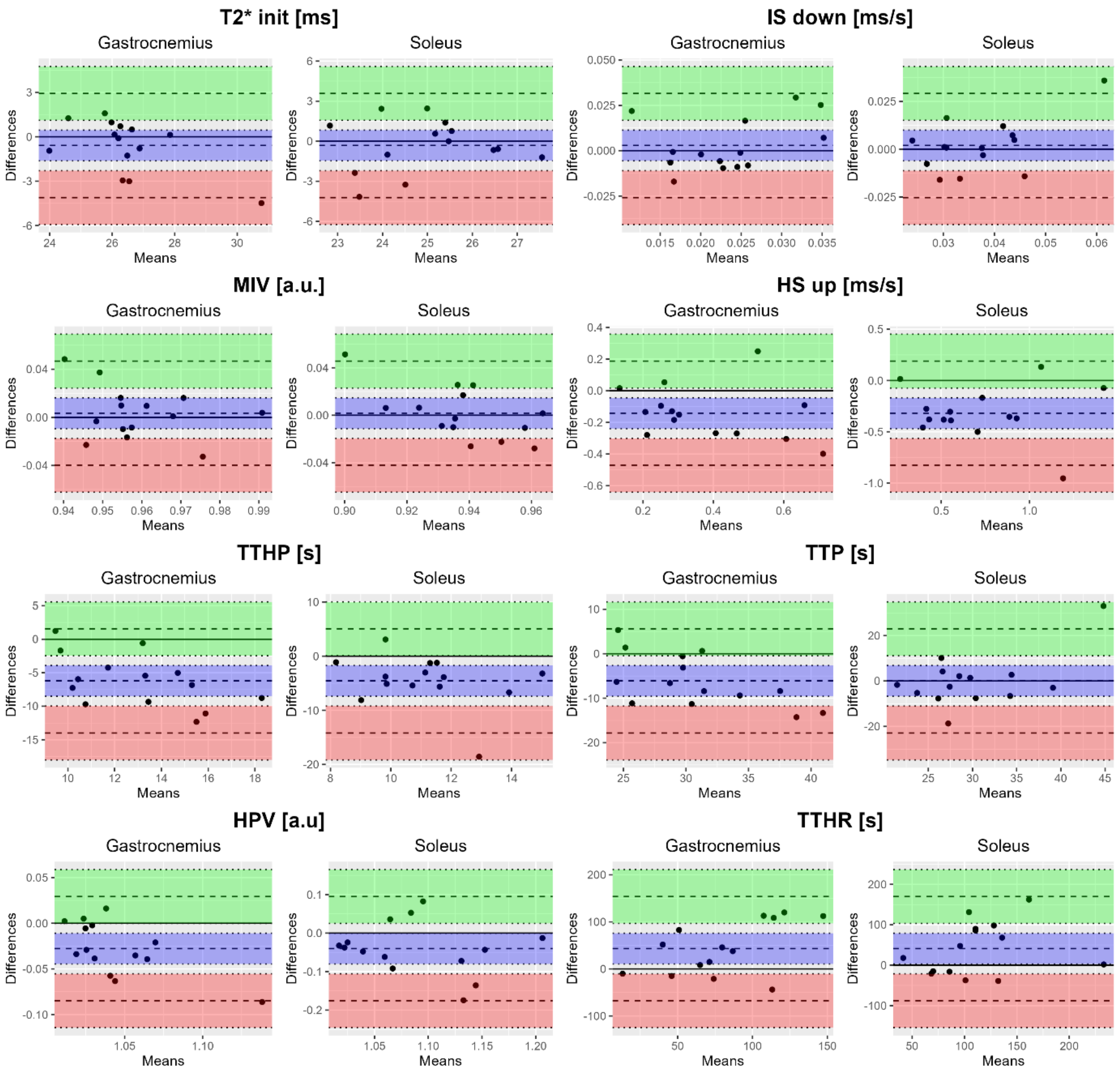

Supporting Figure S1. *Bland-Altman plots illustrating the agreement between the long (5 min) and short (1.5 min) occlusion duration (n=14), evaluated for eight parameters and on two separate muscles. The 95% confidence intervals of estimated lower and upper limits of agreement as well as the bias are colored in red, green, and blue, respectively.*

Intra-session repeatability evaluation

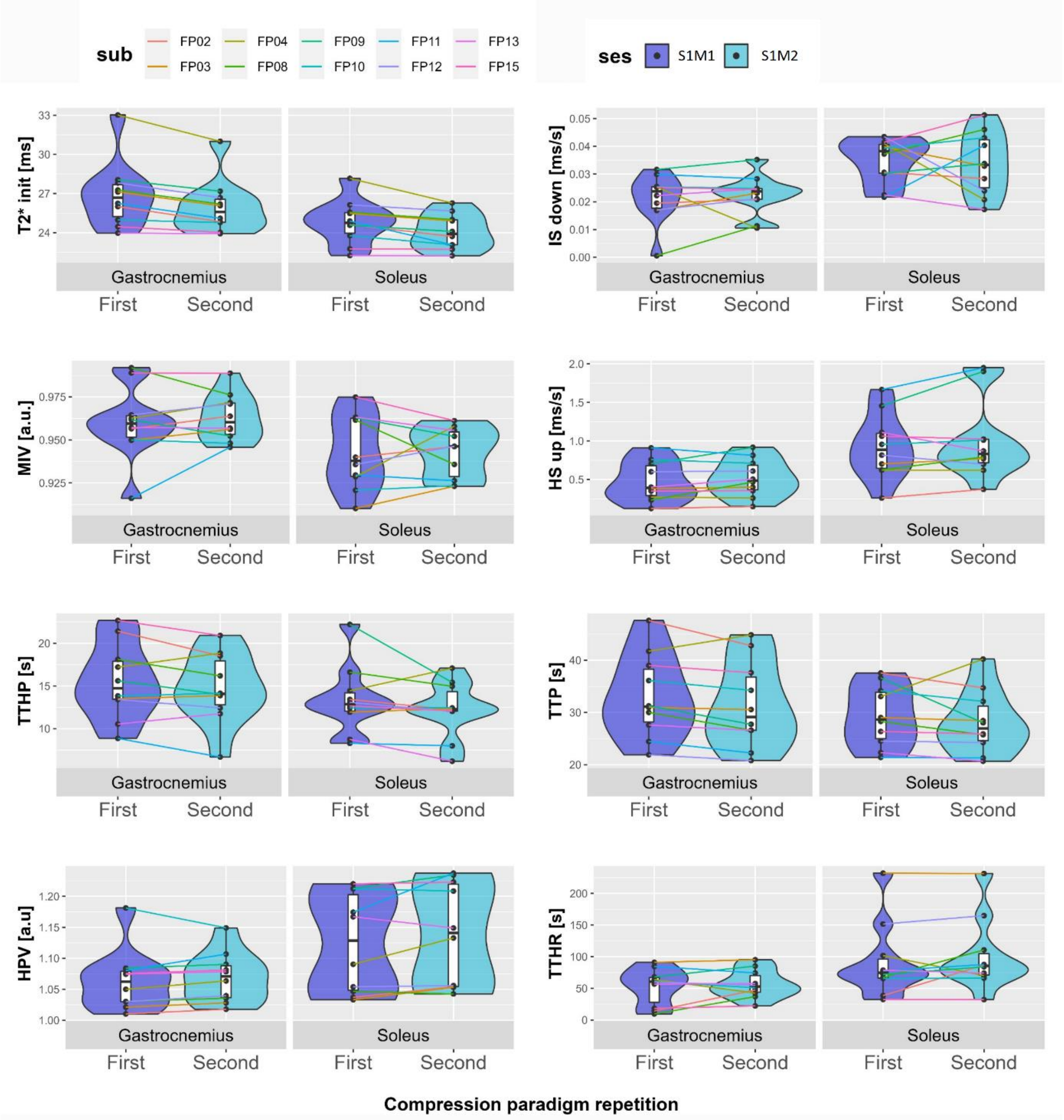

Supporting Figure S2. Violin plots illustrating the intra-session repeatability evaluation (n=10) for the cuffing paradigm with the 5-minute occlusion, evaluated for eight parameters and two muscles. Significance at $p<0.05$ and $p<0.005$ is indicated with a * and ** beside the ROI name for each subset.

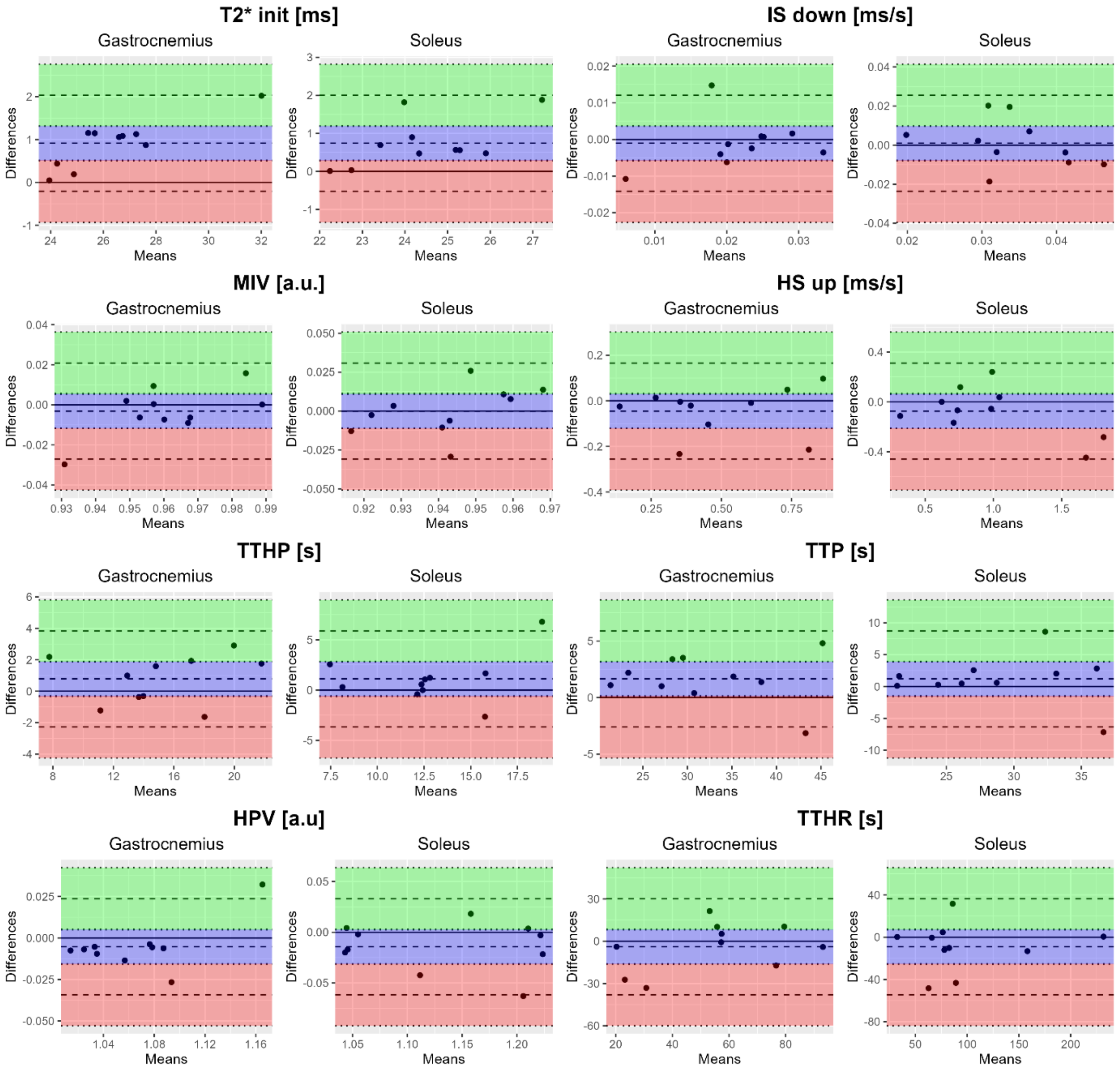

Supporting Figure S3. *Bland-Altman plots illustrating the agreement of intra-session measurements (n=10) for the cuffing paradigm with the 5-minute occlusion, covering eight parameters and two muscles. The 95% confidence intervals of estimated lower and upper limits of agreement as well as the bias are colored in red, green, and blue, respectively.*

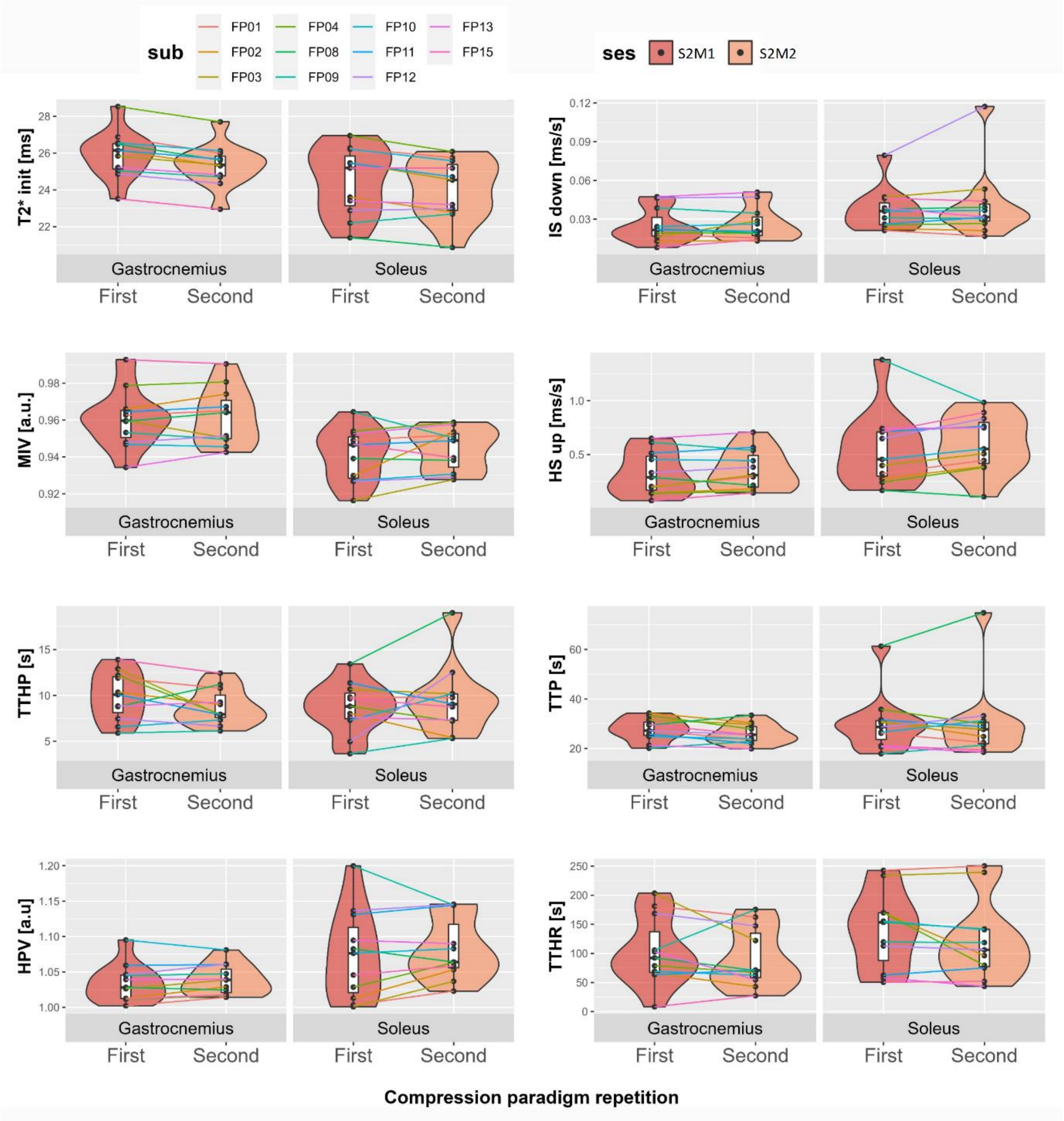

Supporting Figure S4. *Evaluation of repeated occlusions within an imaging session for the 1.5-minute occlusion duration (n=11), evaluated for eight parameters and on two separate muscles. Significance at p<0.05 and p<0.005 is indicated with a * and ** beside the ROI name for each subset.*

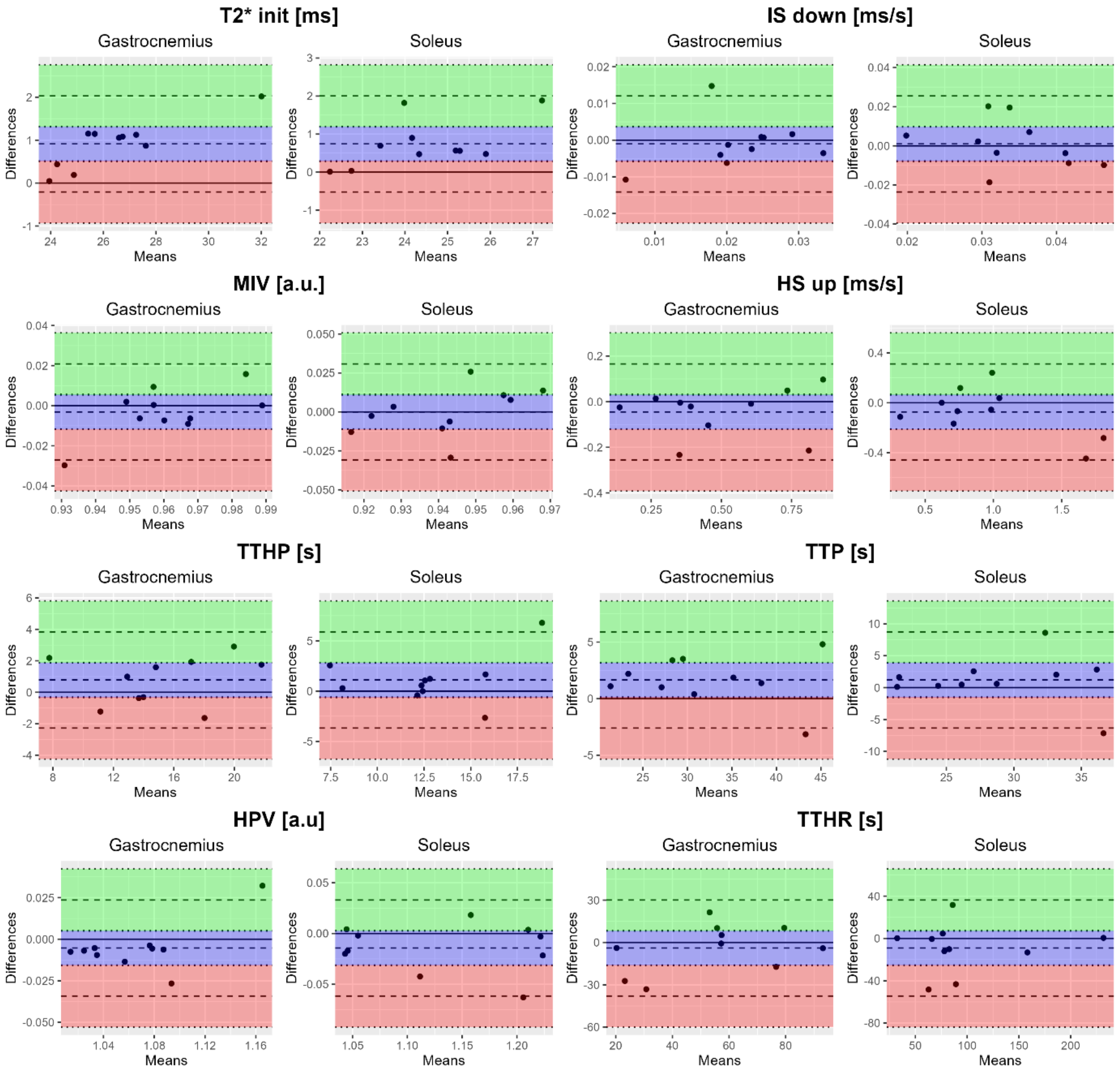

Supporting Figure S5. *Bland-Altman plots illustrating the agreement between repeated occlusions within an imaging session for the 1.5-minute occlusion duration (n=11), evaluated for eight parameters and on two separate muscles. The 95% confidence intervals of estimated lower and upper limits of agreement as well as the bias are colored in red, green, and blue, respectively.*

Inter-session repeatability evaluation

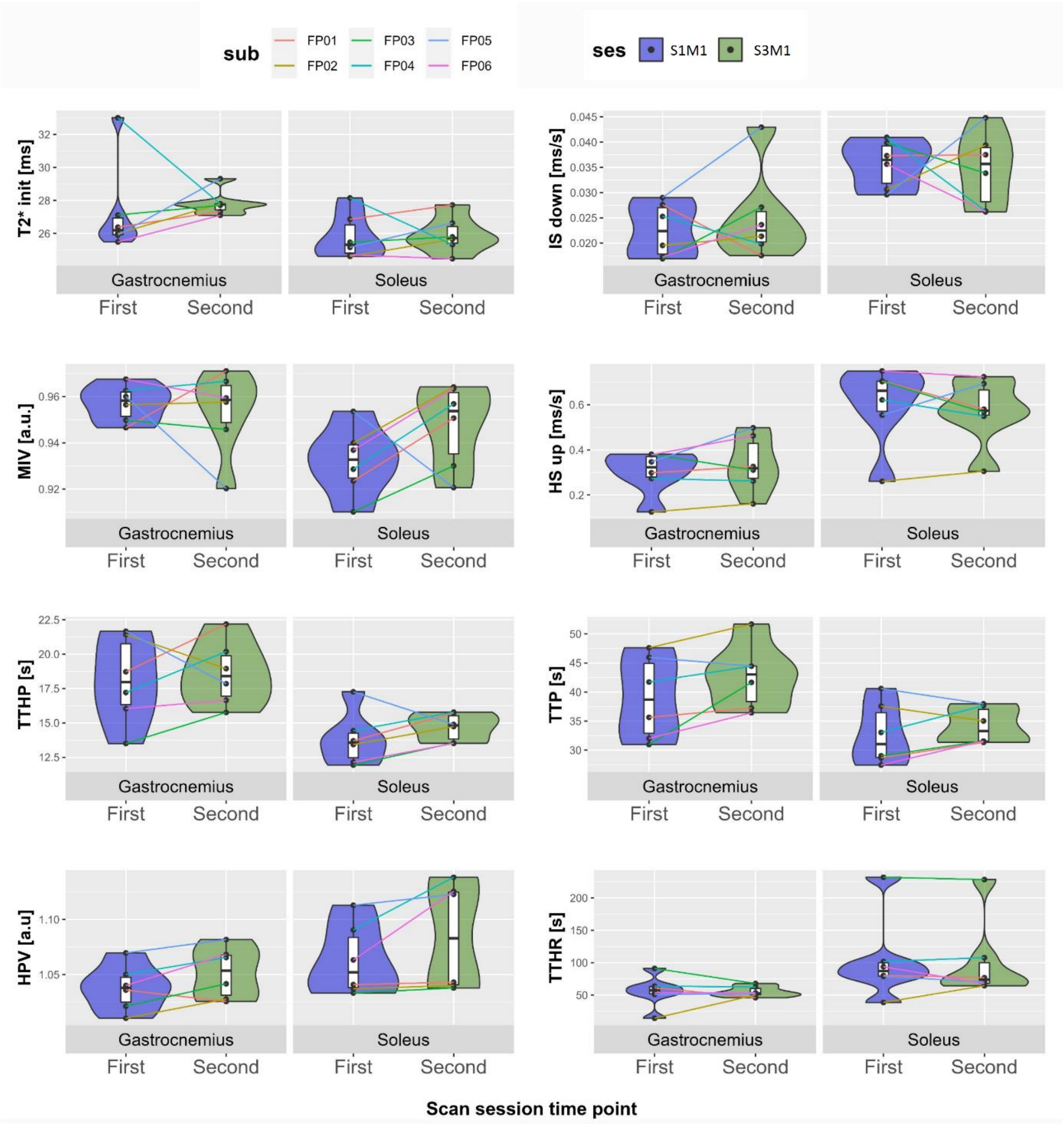

Supporting Figure S6. Violin plots illustrating the inter-session repeatability evaluation (n=6), evaluated for eight parameters and two muscles. Significance at $p<0.05$ and $p<0.005$ is indicated with a * and ** beside the ROI name for each subset.

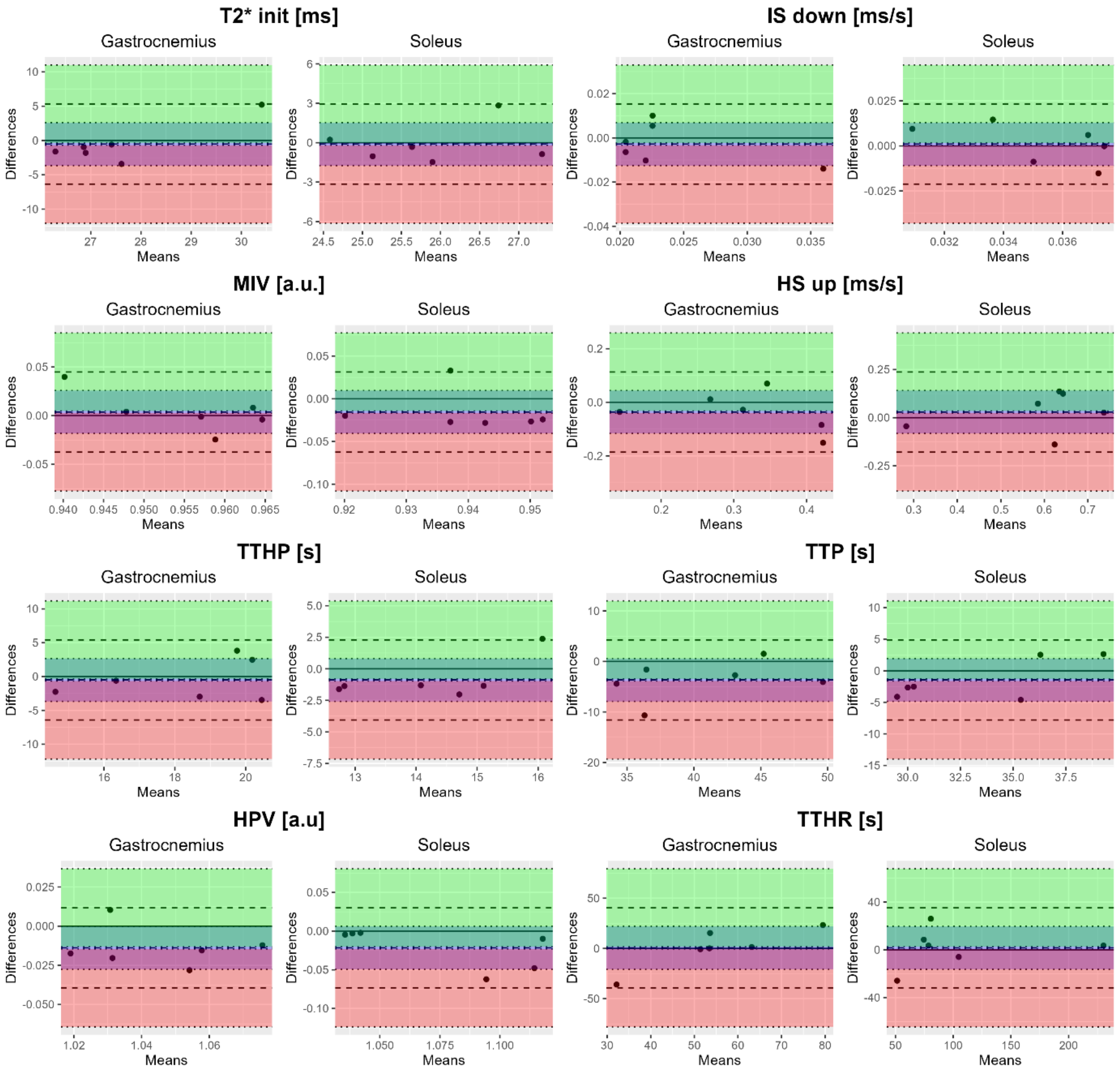

Supporting Figure S7. *Bland-Altman plots illustrating the agreement of inter-session measurements (n=14), evaluated for eight parameters and on two separate muscles. The 95% confidence intervals of estimated lower and upper limits of agreement as well as the bias are colored in red, green and blue, respectively.*

Reproducibility evaluation

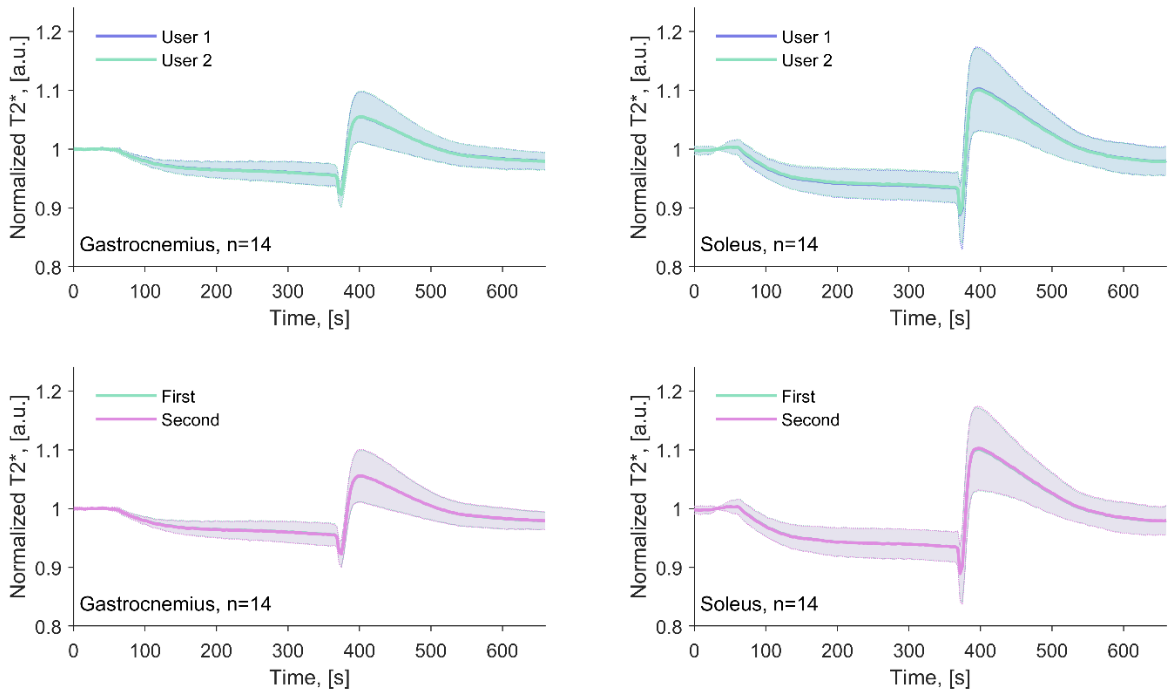

Supporting Figure S8. *Baseline-normalized T2* time curves for interobserver and intraobserver evaluations, displaying the ROI mean time curves as filled lines and ROI standard deviation as transparent area and dotted lines. The gastrocnemius muscles are displayed to the left and soleus muscles to the right.*

Inter-observer reproducibility evaluation

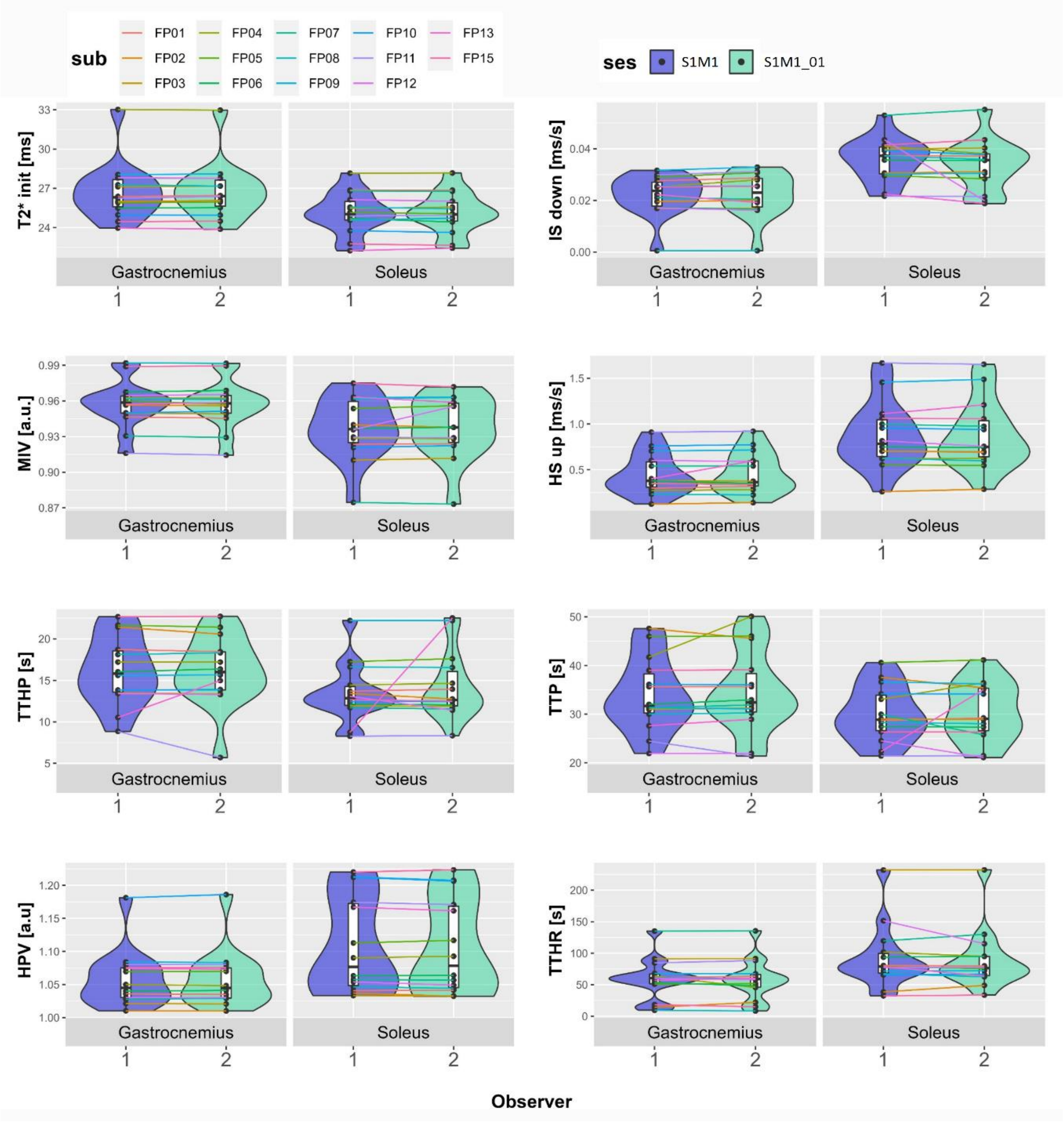

Supporting Figure S9. Violin plots illustrating the inter-observer reproducibility evaluation (n=14), evaluated for eight parameters and two muscles. Significance at p<0.05 and p<0.005 is indicated with a * and ** beside the ROI name for each subset.

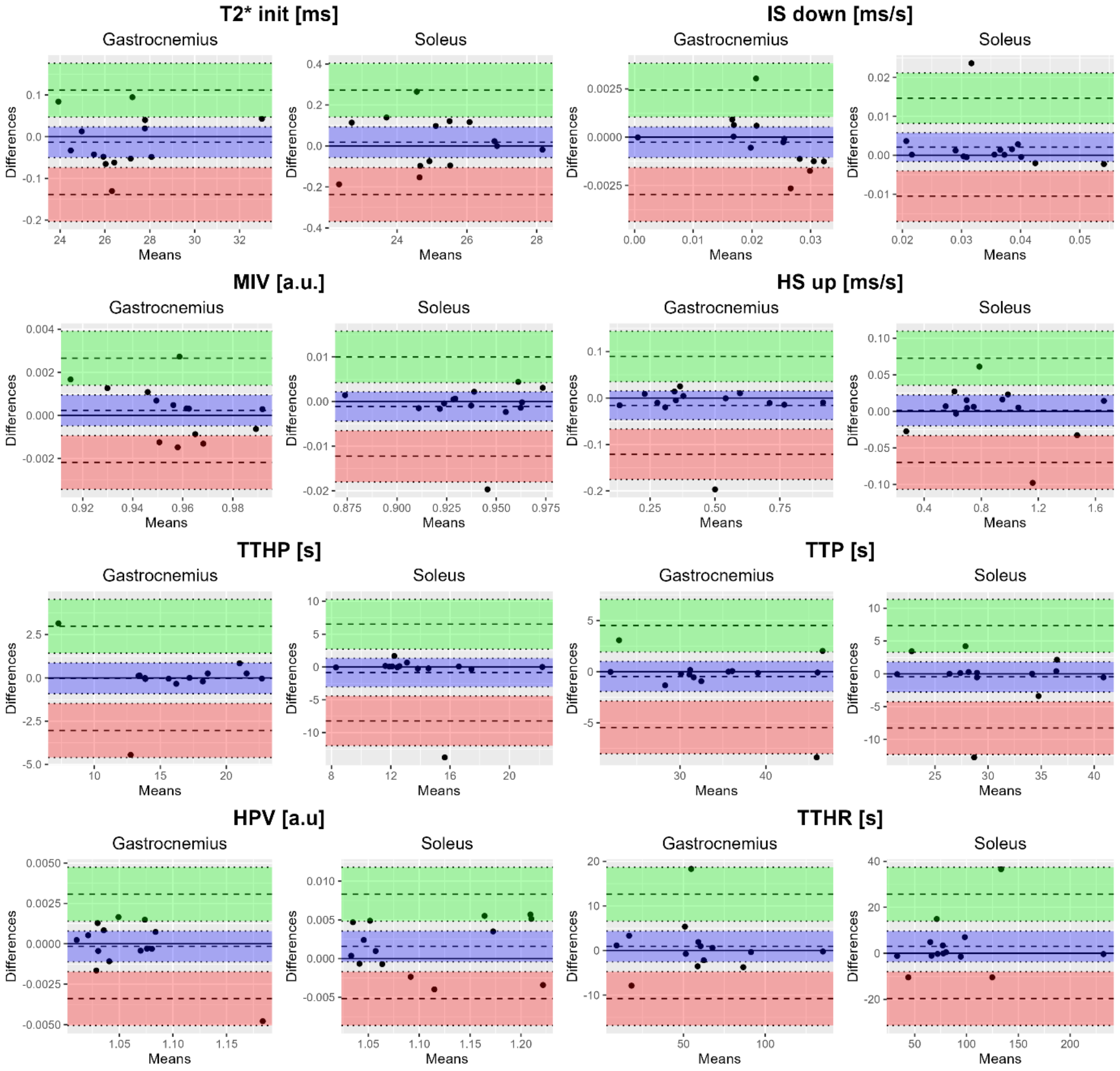

Supporting Figure S10. *Bland-Altman plots illustrating the agreement between* inter-observer *measurements (n=14), evaluated for eight parameters and on two separate muscles. The 95% confidence intervals of estimated lower and upper limits of agreement as well as the bias are colored in red, green, and blue, respectively.*

Intra-observer reproducibility evaluation

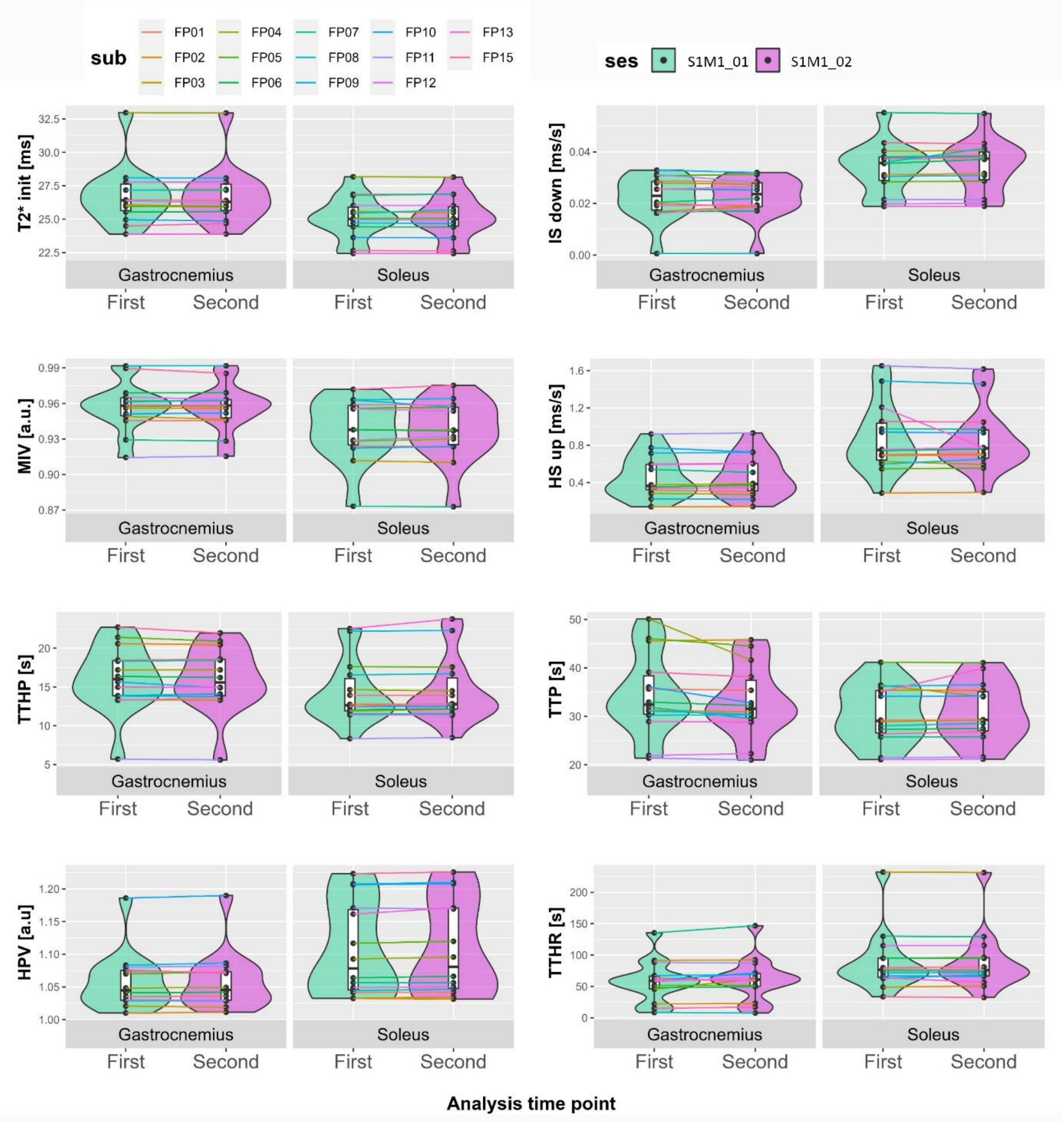

Supporting figure S11. *Violin plots illustrating the intra-observer reproducibility evaluation (n=14), with repeated analysis performed 3 weeks apart and covering eight parameters and two muscles. Significance at p<0.05 and p<0.005 is indicated with a * and ** beside the ROI name for each subset.*

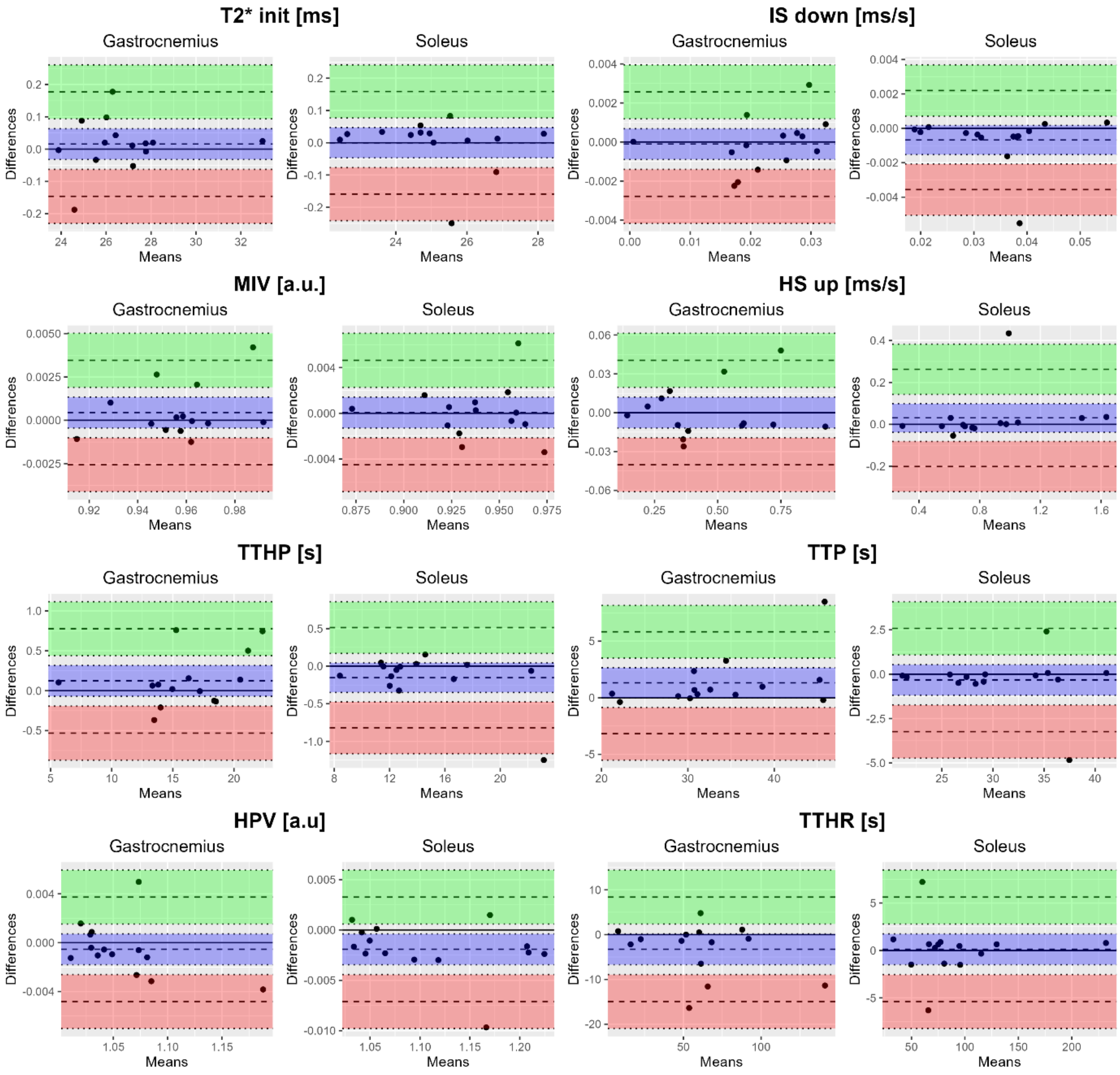

Supporting Figure S12. *Bland-Altman plots illustrating the agreement between intra-observer measurements (n=14), evaluated for eight parameters and on two separate muscles. The 95% confidence intervals of estimated lower and upper limits of agreement as well as the bias are colored in red, green, and blue, respectively.*
